# Efficacy and safety of different systemic drugs in the treatment of uremic pruritus among hemodialysis patients: a network meta-analysis based on randomized clinical trials

**DOI:** 10.3389/fmed.2024.1334944

**Published:** 2024-04-05

**Authors:** Xueqian Zhao, Haipeng Sun, Wei Li

**Affiliations:** Department of Nephrology, The Affiliated Taian City Central Hospital of Qingdao University, Taian, China

**Keywords:** uremic pruritus, hemodialysis, systemic drugs, network meta-analysis, Bayesian

## Abstract

**Aim:**

This network meta-analysis was to analyze and rank the efficacy and safety of different systemic drugs in the treatment of uremic pruritus (UP) among hemodialysis patients.

**Method:**

PubMed, Embase, Cochrane Library, and Web of Science databases were searched from inception to 10 July 2023 for randomized controlled trials (RCTs) investigating different drugs in the treatment of UP among hemodialysis patients. Drugs including cromolyn sodium, dexchlorpheniramine, difelikefalin, gabapentin, hydroxyzine, ketotifen, melatonin, montelukast, nalbuphine, nalfurafine, nemolizumab, nicotinamide, pregabalin, sertraline, thalidomide, and placebo were assessed. Outcome measures, including pruritus relief, response, and adverse events, were analyzed. Network plots, forest plots, league tables, and the surface under the cumulative ranking (SUCRA) probabilities were depicted for each outcome.

**Results:**

The network meta-analysis retrieved 22 RCTs. Gabapentin (69.74%) had the highest likelihood to be the most effective drug for pruritus relief in UP patients receiving hemodialysis, followed by cromolyn sodium and hydroxyzine. Thalidomide (60.69%) and gabapentin (58.99%) were associated with significantly more drug responses for treating UP among patients receiving hemodialysis. Patients who were treated with gabapentin (40.01%) were likely to have risks of adverse events and dizziness. Lower risks of adverse events, nausea, and diarrhea were found in patients who received cromolyn sodium and lower risks of somnolence.

**Conclusion:**

This study suggests considering gabapentin treatment when facing a patient suffering from UP. This study provides a reference for the selection of drug therapy for UP patients receiving hemodialysis.

## 1 Introduction

Uremic pruritus (UP), also known as chronic kidney disease-associated pruritus (CKD-aP), is a common, bothersome, and sometimes debilitating symptom in patients with CKD or end-stage renal disease (ESRD) (1, 2). The incidence of UP in the literature varied from 22 to 84% of patients undergoing hemodialysis, and approximately 20–40% of patients reported moderate-to-severe pruritus (3, 4). UP is strongly associated with physical and mental limitations, insomnia, and chronic fatigue; discomfort, embarrassment, and isolation; and secondary skin changes from scratching lesions; anger, anxiety, and depression (5, 6). UP patients receiving dialysis also reported higher all-cause mortality, infection-related mortality, and cardiovascular-related mortality (7, 8). There is, therefore, a need for effective interventions for the treatment of UP.

Several therapeutic interventions have been studied to improve UP with variable degrees of success, including optimizing dialysis (remove uremic toxins), correcting Ca-Ph-parathormone abnormalities, gabapentin and pregabalin (modulating neuropathic pain), montelukast (leukotriene inhibition), naltrexone and nalfurafine (targeting μ and κ receptors), difelikefalin (agonist of the κ-opioid receptor), sertraline (selective serotonin reuptake inhibitors), emollients, supplements, and phototherapy (2, 6, 9, 10). These UP treatments were separated into three main groups: systemic, topical, and other complementary alternative medicines (6). Initial treatment involves topical therapy, mainly in the form of moisturizers; however, in many cases, this is not sufficient to relieve itching (11). In the literature review, the author concluded that new treatments still need large population studies and further exploration (6). For systemic drugs, their efficacy has already been supported by large-scale clinical evidence. In a double-blinded, placebo-controlled, multi-centric randomized clinical trial (RCT), UP patients treated with sertraline had a significant improvement in pruritus as compared with those who received placebo (2). According to a phase 2 study, oral difelikefalin significantly reduced itch intensity in stage 3–5 CKD subjects with moderate-to-severe pruritus (12). In another RCT, oral nalfurafine effectively reduced itching in Chinese hemodialysis patients with refractory pruritus (13). An RCT by Gobo-Oliveira et al. reported that UP was reduced upon treatment with gabapentin or dexchlorpheniramine with good safety profiles; however, no difference was observed between the two treatments (11). Considering the inconsistency in drug selection and the lack of guidelines to guide drug selection for UP in clinical practice, a meta-analysis was necessary to assess the efficacy of multiple systemic drugs for the treatment of UP in hemodialysis patients. In addition, systemic drugs may be associated with adverse effects. Somnolence and dizziness are common side effects of gabapentin (14). However, in a pooled analysis from the phase 3 clinical trial program, intravenous difelikefalin demonstrated an acceptable safety profile and was generally well tolerated with long-term use for the treatment of moderate-to-severe pruritus in hemodialysis patients (15). In an RCT, nalfurafine was found to be a safe treatment option for Chinese hemodialysis patients with pruritus (13). Given the inconsistent results, the adverse risks of systemic drugs for UP must be monitored carefully.

Herein, the purpose of this network meta-analysis was to analyze and rank the efficacy and safety of different drugs in the treatment of UP among hemodialysis patients, so as to provide a reference for the treatment selection of UP in hemodialysis patients.

## 2 Methods

This network meta-analysis was reported in accordance with the preferred reporting items for systematic reviews and meta-analyses (PRISMA) and the PRISMA extension statement for network meta-analysis (16).

### 2.1 Search strategy

From inception to 10 July 2023, PubMed, Embase, Cochrane Library, and Web of Science databases were searched using the following terms: “Uremia*” OR “Uremic” OR “Chronic Kidney Disease” OR “CKD” OR “Chronic Kidney Failure” OR “Chronic Renal Failure” OR “End Stage Kidney Disease” OR “End Stage Renal Disease” OR “ESRD” OR “End Stage Renal Failure” OR “ESRF” OR “Renal Dialysis” OR “Renal Dialyses” OR “Hemodialysis” OR “Hemodialyse” OR “Extracorporeal Dialysis” OR “Extracorporeal Dialyses” OR “Hemodiafiltration” OR “Hemofiltration” OR “Advanced Renal Disease” AND “Pruritus” OR “Pruritis” OR “Itch.”

### 2.2 Inclusion and exclusion criteria

The inclusion criteria were implemented based on the Population, Intervention, Comparison, Outcomes, and Study (PICOS) design framework: (1) population (P): UP patients undergoing hemodialysis; (2) interventions (I) and controls (C): studies compared the effectiveness of various treatments including cromolyn sodium, dexchlorpheniramine, difelikefalin, gabapentin, hydroxyzine, ketotifen, melatonin, montelukast, nalbuphine, nalfurafine, nemolizumab, nicotinamide, pregabalin, sertraline, thalidomide, and placebo; (3) outcomes (O): pruritus relief, response, and adverse events; (4) study design (S): RCTs; (5) literature published in English; (6) for different studies reporting on the same population, only the most recent or largest sample sizes were included.

The exclusion criteria were: (1) animal experiments; (2) patients were mixed population; (3) interventions included topical drug therapy or supplements, such as capsaicin cream, emollient, tacrolimus ointment, baby oil, and gamma-linolenic acid; (4) studies with incomplete data, inability to extract data, or inability to access network graph connectivity; (5) case reports, conference abstracts, letters, erratum, protocols, reviews, and meta-analyses.

### 2.3 Data extraction and quality assessment

Data were extracted from included studies, including the first name of the author, year of publication, country, study design, sample size, sex, age, pruritus score, duration of pruritus, pruritus assessment, duration of hemodialysis, primary diseases, drugs in use, dosage, duration of treatment, and outcomes.

The quality of RCTs was assessed by the Cochrane risk bias assessment tool (17), which mainly divides bias into the following six domains: selection bias, implementation bias, measurement bias, follow-up bias, reporting bias, and other bias. Each domain was measured as low bias, unclear bias, or high bias. The Grading of Recommendations Assessment, Development, and Evaluation (GRADE) Working Group (18) has developed a sensible and transparent approach to grading the quality of evidence in the network analysis, and the evidence is evaluated based on five domains: study limitations (risk of bias), inconsistency and heterogeneity, indirectness, imprecision, and publication bias. The overall quality was considered to be high if multiple RCTs with a low risk of bias provided consistent, generalizable results for the outcome. The GRADE method categorizes the quality of evidence into four levels as follows: very low, low, moderate, and high. Data extraction and quality assessment were independently carried out by two authors (Xueqian Zhao and Haipeng Sun); if consensus could not be reached, a third reviewer (Wei Li) was included to resolve disputes.

### 2.4 Outcomes assessment

In our study, we standardized the assessment of pruritus on a 0–10 scale (pruritus score in Table 1) based on a visual analog scale (VAS) or numeric rating scale (NRS) (2, 12), with higher scores indicating more severe itching. Pruritus relief was defined based on the change in pruritus score from pre-treatment to post-treatment, with a greater difference indicating more pronounced relief. The response was defined as 3-point improvement in the NRS score or a 50% or more reduction in pruritus values. The adverse outcomes included nausea, diarrhea, somnolence, and dizziness.

**Table 1:** Basic characteristics of the included studies

Author	Year	Country	Design	Groups	Sample size(N)	Sex (Male/Female)	Age (years)	Pruritus score	Pruritus duration (years)	Duration of hemodialysis (years)	Primary disease (N)	Drugs in use (N)	Route, dosage	Pruritus assessment	Treatment duration (weeks)	Outcomes
Elsayed	2023	Egypt	DBRCT	Sertraline	30	10/20	43.67 ± 12.63	5.27 ± 2.75	1.70 ± 1.24	4.69 ± 4.34	Hypertension 14, DM 7, PKD 2, GN 5, others 2	Excluded patients consumed antihistamines, opioid antagonists, immunosuppressants, cholestyramine, corticosteroids, ultraviolet B phototherapy or emollients cream 1 month before study	Oral, 50mg twice daily	VAS based on self-reported measures of symptoms, had five categories of severity, 0 (no pruritus), <3 (mild), ≥3-<7 (moderate), ≥7-<9 (severe), ≥9 (very severe)	8	Pruritus relief, nausea, diarrhea
				Placebo	30	13/17	50.27 ± 13.09	4.57 ± 1.94	1.92 ± 1.50	4.12 ± 3.38	Hypertension 16, DM 3, PKD 1, GN 9, others 1		Oral, twice daily			
Yosipovitch	2023	USA	DBRCT	Difelikefalin	202	102/100	67.3 ± 11.6	7.1 ± 1.2	-	-	-	Gabapentinoids 43, antihistamines 30, topical corticosteroids 7	Oral, 0.25 0.5 or 1.0 mg once daily	WI-NRS scores range from 0 (no itching) to 10 (worst itching imaginable)	12	Pruritus relief
				Placebo	67	37/30	65.6 ± 12.1	7.0 ± 1.1				Gabapentinoids 18, antihistamines 7, topical corticosteroids 6	Oral, once daily			
Zhang	2023	China	DBRCT	Nalfurafine	114	86/28	55.45 ± 13.43	8.25 ± 1.02	2.54 ± 2.88	5.76 ± 3.74	GN 49, DN 22, others 43	Calcium channel blockers 84, β-blockers 77, RAAS inhibitors 60, diuretics 8, topical treatment 88, antihistamines 27, anti-allergic 3, gabepentin 4	Oral, 5 or 2.5 μg once daily	VAS consisted of a 100 mm horizontal line measured in millimeters with no scale markings, the left end of the line (0 mm) represented no itching and the right end (100 mm) the worst itching imaginable	2	Adverse events, nausea
				Placebo	27	21/6	56.6 ± 14.51	8.23 ± 0.85	2.97 ± 3.61	5.2 ± 3.63	GN 10, DN 10, others 7	Calcium channel blockers 18, β-blockers 15, RAAS inhibitors 13, diuretics 1, topical treatment 23, antihistamines 5, anti-allergic 1	Oral, once daily			
Fishbane	2022	multiple countries	DBRCT	Difelikefalin	424	247/177	59.0 ± 12.3	-	2.1 (3.2)#	3.5 (4.8)#	-	Diphenhydramine 104, hydroxyzine 42, hydrocortisone 11, cetirizine 7, clemastine 7	IV, 0.5 mcg/kg thrice weekly	-	12	Adverse events, nausea, diarrhea, somnolence, dizziness
				Placebo	424	257/167	58.4 ± 13.5		2.5 (3.2)#	3.9 (5.0)#		Diphenhydramine 100, hydroxyzine 52, hydrocortisone 14, cetirizine 10, clemastine 10	IV, thrice weekly			
Narita	2022	Japan	DBRCT	Difelikefalin	183	142/41	64.7 ± 11.4	6.55 ± 1.32	4.3 ± 4.3	7.1 ± 6.7	DN 92, chronic GN 36, nephrosclerosis 29, PKD 6, other 12, unspecified 12	Corticosteroids 74, antihistamines 139, moisturizers 116, others 51	IV, 0.25 0.5 or 1.0 μg/kg thrice weekly	WI-NRS scores range from 0 (no itching) to 10 (worst itching intensity)	8	Response, adverse events, nausea, somnolence, dizziness
				Placebo	63	43/20	64.1 ± 12.7	6.53 ± 1.31	4.3 ± 4.4	6.8 ± 6.1	DN 27, chronic GN 10, nephrosclerosis 10, PKD 3, other 8, unspecified 8	Corticosteroids 21, antihistamines 51, moisturizers 33, others 19	IV, thrice weekly			
Topf	2022	multiple countries	DBRCT	Difelikefalin	426	249/177	59.1 ± 12.4	7.2 ± 1.4	2.1 (3.2)#	3.5 (4.8)#	Diabetes 225, hypertension 122, GN 18, cystic kidney 14, other 47	Diphenhydramine 104, hydroxyzine 42, hydrocortisone 11, cetirizine 7, clemastine 7	IV, 0.5 mcg/kg thrice weekly	WI-NRS scores range from 0 (no itching) to 10 (worst itching imaginable)	12	Response
				Placebo	425	258/167	58.3 ± 13.5	7.2 ± 1.5	2.5 (3.2)#	3.9 (5.0)#	Diabetes 206, hypertension 138, GN 16, cystic kidney 15, other 50	Diphenhydramine 100, hydroxyzine 52, hydrocortisone 16, cetirizine 10, clemastine 10	IV, thrice weekly			
Baharvand	2021	Iran	DBRCT	Melatonin	23	7/16	54.52 ± 13.00	8.56 ± 2.00	-	3.89 ± 4.06	Hypertension 15, DM 13, others 4	Gabapentin 5, sertraline 1, ketotifen 1	Oral, 5 mg once daily	VAS consisted of 10-cm long line (oriented horizontally or vertically), beginning of the scale refers to no pruritus (0 points) and the end to the most severe pruritus they can imagine (10 points)	2	Pruritus relief
				Placebo	16	14/2	55.88 ± 11.70	6.63 ± 1.93		10.00 ± 10.13	Hypertension 8, DM 5, others 9	Gabapentin 3, hydroxyzine 1	Oral, once daily			
Kinugasa	2021	Japan	DBRCT	Nemolizumab	41	33/8	58.3 ± 8.8	6.56 ± 1.07	5.2 ± 5.3	6.9 ± 5.6	DN 20, chronic GN 8, nephrosclerosis 6, PKD 5	-	SC, 0.125 0.5 or 2.0 mg/kg on day 1	VAS scores on a scale of 0 (no itch) to 10 (worst imaginable itch)	4	Adverse events, diarrhea
				Nalfurafine	12	10/2	58.3 ± 13.0	6.98 ± 1.33	7.2 ± 5.6	8.5 ± 6.4	DN 5, chronic GN 4, nephrosclerosis 2		Oral, 2.5–5 μg once daily	VAS scores on a scale of 0 (no itch) to 10 (worst imaginable itch)		
				Placebo	14	10/4	55.1 ± 11.1	6.93 ± 1.24	4.2 ± 4.4	7.9 ± 5.1	DN 5, chronic GN 4, nephrosclerosis 3		SC, on day 1	VAS scores on a scale of 0 (no itch) to 10 (worst imaginable itch)		
Fishbane	2020	USA	DBRCT	Difelikefalin	129	77/52	26-84	6.8±1.4	4.4 ± 3.9	5.7 ± 4.7	Diabetes 63, hypertension and large-vessel disease 65, GN/vasculitis 12, other 7, cystic/hereditary/congenital disease 5, urologic 1, unknown 1	Any prior anti-pruritic medication 55, diphenhydramine hydrochloride 33, hydroxyzine hydrochloride 11, topical hydrocortisone 4	IV, 0.5 1.0 or 1.5 μg/kg thrice weekly	WI-NRS scores range from 0 (no itching) to 10 (worst itching imaginable)	8	Response, adverse events nausea, diarrhea, somnolence, dizziness
				Placebo	45	28/17	60 (27-87)	6.8 ± 1.5	4.4 ± 4.7	5.9 ± 4.9	Diabetes 21, hypertension and large-vessel disease 21, GN/vasculitis 5, other 1, interstitial nephritis/ pyelonephritis 1	Any prior anti-pruritic medication 18, diphenhydramine hydrochloride 11, hydroxyzine hydrochloride 2, topical hydrocortisone 5	IV, thrice weekly	WI-NRS scores range from 0 (no itching) to 10 (worst itching imaginable)		
Kebar	2020	Iran	DBRCT	Gabapentin	16	8/8	58.2 ± 8.1	7.10 ± 1.46	-	-	-	0	Oral, 100 mg/d	VAS	6	Pruritus relief
				Hydroxyzine	16	8/8	56.7 ± 7.6	6.83 ± 2.11					Oral, 25 mg/d			
Ravindran	2020	India	single-blind RCT	Pregabalin	21	14/7	55.29 ± 14.58	-	-	-	-	-	Oral, 25 mg	VAS	6	Adverse events
				Gabapentin	21	17/4	58.10 ± 11.09						Oral, 100 mg			
Gobo Oliveira	2018	Brazil	DBRCT	Gabapentin	30	15/15	64 ± 15	5 (4-8)*	0.7 (0.3-2.0)*	3.0 (1.0-4.3)*	DM 9, hypertension 7, GN 2, others 12	-	Oral, 300 mg thrice weekly	VAS up to 10 levels	3	Adverse events
				Dexchlorpheniramine	30	19/11	59 ± 12	5 (3-7)*	0.5 (0.3-2.0)*	2.5 (1.1-4.0)*	DM 11, hypertension 4, GN 3, others 12		Oral, 6 mg twice daily			
Mahmudpour	2017	Iran	DBRCT	Montelukast	40	-	53.3 ± 15.8	6.43 ± 2.36	-	-	-	Medications with antipruritic effects were discontinued 1 week before the study	Oral, 10 mg daily	VAS	4	Pruritus relief
				Placebo	40			6.00 ± 1.94					Oral, daily			
Mathur	2017	USA, Romania, Poland	DBRCT	Nalbuphine	248	112/136	55 ± 12	6.9 ± 1.4	3.2 ± 2.9	4.8 ± 4.1	-	Antihistamines 78, corticosteroids 17, gabapentin 2	Oral, 120 or 60 mg twice daily	NRS consisted of an 11-point scale, 0 (no itching) to 10 (worst possible itching)	7	Pruritus relief, nausea, somnolence
				Placebo	123	59/64	57 ± 13	6.8 ± 1.4		4.5 ± 4.4			Oral, twice daily			
Amirkhanlou	2016	Iran	DBRCT	Gabapentin	26	12/14	60.2 ± 7.4	-	-	-	-	-	Oral, 100 mg daily	0= no itching, 1= minimal, 2= mild, 3= moderate and 4= severe itching	2	Adverse events, somnolence, dizziness
				Ketotifen	26	13/13	57.6 ± 6.2						Oral, 1 mg twice daily			
Nofal	2016	Egypt	single-blind RCT	Gabapentin	27	23/4	51.50 ± 9.96	7.63 ± 2.00	5.15 ± 3.89	7.62 ± 4.53	-	Any medications with antipruritic effects were discontinued one week before the study	Oral, 100-300 mg thrice weekly	VAS consists of a 10 cm horizontal line marked from zero (no itch) to 10 (worst possible itch)	4	Pruritus relief, response, adverse events, somnolence, dizziness
				Placebo	27	18/9	52.15 ± 9.94	6.90 ± 1.97	4.98 ± 3.74	5.66 ± 3.37			Oral, thrice weekly			
Omidian	2013	Iran	DBRCT	Nicotinamide	24	-	49.6 ± 12.7	5.44 ± 0.74		3.7	-	0	Oral, 500 mg twice daily	VAS numbers from 0 to 5 (0: No pruritus and 5: The worst pruritus)	4	Pruritus relief
				Placebo	25			5.92 ± 0.90					Oral, twice daily			
Kumagai	2010	Japan	DBRCT	Nalfurafine	226	178/48	60.3 ± 11.4	6.7 ± 1.4	-	-	-	Topical agents 49, oral antihistamines 55, antihistamine injection 13, oral anti-allergy 142, hypnotics or anxiolytics 89, antiepileptics 9, antipsychotics or antidepressants 38	Oral, 5 or 2.5 μg once daily	VAS consisting of a 100-mm horizontal line with no scale markings, strongest possible itch marked at the right end of the line (100 mm) and no itch marked at the left end (0 mm)	2	Response, adverse events, diarrhea, somnolence
				Placebo	111	89/22	59.6 ± 11.8	6.5 ± 1.4				Topical agents 26, oral antihistamines 26, antihistamine injection 6, oral anti-allergy 75, hypnotics or anxiolytics 35, antiepileptics 3, antipsychotics or antidepressants 15	Oral, once daily	VAS consisting of a 100-mm horizontal line with no scale markings, strongest possible itch marked at the right end of the line (100 mm) and no itch marked at the left end (0 mm)		
Vessal	2010	Iran	DBRCT	Cromolyn_ sodium	21	12/9	56.90 ± 15.49	8.48 ± 2.02	-	2.64 ± 1.70	-	Any medication that had antipruritic effect was discontinued 1 week before the study	Oral, 135 mg thrice daily	VAS range from 0 (absence of pruritus) to 10 (the greatest severity of symptoms)	8	Pruritus relief, adverse events, nausea, diarrhea
				Placebo	19	8/11	57.47 ± 13.6	8.68 ± 1.82		2.61 ± 2.20			Oral, thrice daily	VAS range from 0 (absence of pruritus) to 10 (the greatest severity of symptoms)		
Naini	2007	Iran	DBRCT	Gabapentin	17	16/18	62 ± 10	7.2 ± 2.3	-	-	-	Any medication with presumed antipruritic effects was discontinued one week before the study	Oral, 400 mg twice weekly	VAS consisting of a 10 cm horizontal line marked from zero (no itch) to 10 (worst possible itch)	4	Pruritus relief, dizziness
				Placebo	17								Oral, twice weekly			
Wikstrom	2005	Japan, Sweden, Poland, Denmark	DBRCT	Nalfurafine	42	-	≥ 18	6.47 ± 1.36	-	-	-	All anti-pruritic medications, except for topical neutral agents, were discontinued for at least 7 d	IV, 5 μg thrice weekly	VAS consisted of a 100 mm horizontal line measured in millimeters, the left end of the line (0 mm) represented no itching and the right end (100 mm) the worst itching ever	4	Pruritus relief, Response, adverse events
				Placebo	43			6.39 ± 1.40					IV, thrice weekly			
Silva	1994	Brazil	DBRCT	Thalidomide	14	12/2	57.5 ± 7.3	5.87 ± 0.65	-	5.4 ± 3.2	Chronic GN 1, malignant nephrosclerosis 6, others 7	Antihypertensive drugs 10, phosphate binders 17, calcitriol 10, H-blockers 3, erythropoietin 2, iron supplements 6, vitamins 12	Oral, 100 mg	0, absent; 1 pruritus at rest or during usual tasks but not interfering with its accomplishment; 2, pruritus perturbing but not interrupting performance of regular tasks; 3, pruritus causing interruption of tasks or sleep	1	Response
				Placebo	15	5/10	50.5 ± 11.2	-		5.1 ± 3.5	Chronic GN 4, malignant nephrosclerosis 5, PKD 3, others 3		Oral			

Notes: DBRCT, double blinded randomized controlled trial; DM, diabetes mellitus; DN, diabetic nephropathy; IV, intravenous; GN, glomerulonephritis; PKD, polycystic kidney disease; RCT, randomized controlled trial; SC, subcutaneous; VAS, visual analog scale; WI-NRS, worst itching intensity numeric rating scale; #median (IQR), *median (p25-p75).

### 2.5 Statistical analysis

Statistical analysis was conducted in Stata 15.1 software, Revman 5.4, and the Gemtc 1.0.1 package in R 4.1.3 software. The network meta-analysis model was implemented in the Bayesian framework and estimated using Markov chain Monte Carlo (MCMC) methods, which were simulated by four chains; the number of iterations was 20, 000; the number of continued iterations was 50,000; and the step size was set to 1.

Statistical heterogeneity was tested using the *I*^2^ statistic, with values of <25, 25–50%, and > 50% indicating low, moderate, and high heterogeneity, respectively. Consistency, another crucial assumption for network meta-analysis, refers to the statistically consistent results between direct and indirect effect sizes regarding the same comparison. The deviance information criterion (DIC) was used to assess the inconsistency of direct and indirect evidence of the treatment network, where the smaller DIC value was considered to have better consistency. If the difference was within 5, it indicated that the data basically met the premise of consistency.

For the outcomes of pruritus relief, weighted mean difference (WMD) and 95% credible intervals (95% CrIs) for different drugs were reported; for response, adverse events, nausea, diarrhea, somnolence, dizziness, relative risk (RR) values, and 95% CrIs for different drugs were calculated. The network plot for each outcome indicator was drawn. WMD or RR values and 95% CrIs for all direct and indirect comparisons were presented in the forest plot. The ranking probability table was used to rank the pros and cons of the intervention (the value indicates the probability of the intervention at the nth position). We ranked the comparative effects of all drugs with the surface under the cumulative ranking (SUCRA) probabilities.

## 3 Results

### 3.1 Characteristics of included studies

**Figure 1:**
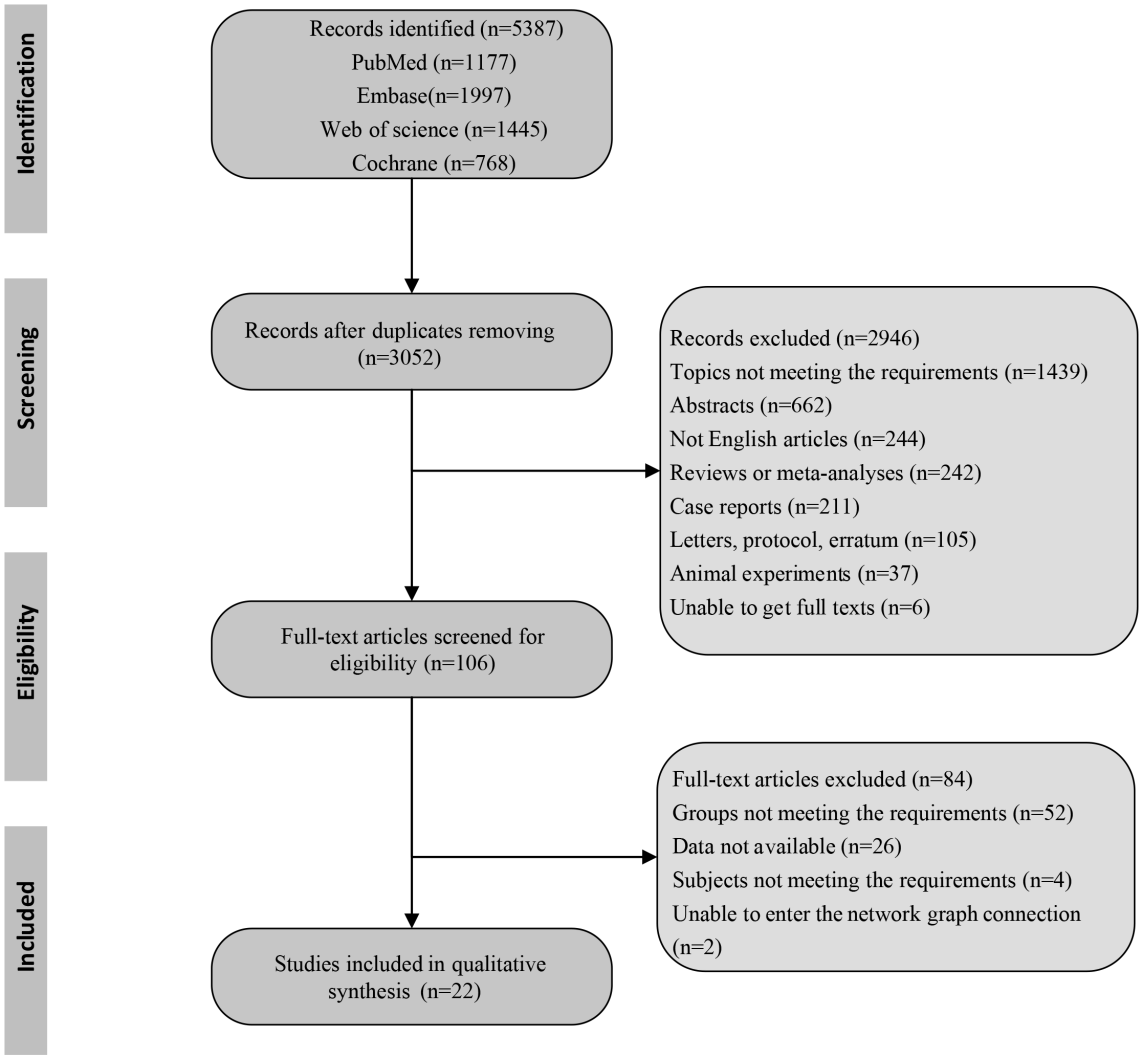
The flow diagram of the study selection.

After searching the database according to the search strategy, a total of 5,387 articles were identified, including 1,177 studies from PubMed, 1,997 studies from Embase, 1,445 studies from Web of Science, and 768 studies from Cochrane. In total, 3,052 records were left after duplicates were removed, and 106 full-text articles were screened for eligibility. Finally, 22 studies (2, 11–13, 15, 19–35) were included in this network meta-analysis. The flow diagram of the study selection is depicted in Figure 1. A total of 2,877 UP patients receiving hemodialysis patients were included, of which 21 patients were treated with cromolyn sodium, 30 patients were treated with dexchlorpheniramine, 763 patients were treated with difelikefalin, 137 patients were treated with gabapentin, 16 patients were treated with hydroxyzine, 26 patients were treated with ketotifen, 23 treated with melatonin, 40 patients received montelukast, 248 patients treated with nalbuphine, 394 patients received nalfurafine, 41 patients received nemolizumab, 24 patients underwent nicotinamide, 1,049 patients treated with placebo, 21 patients received pregabalin, 30 patients treated with sertraline, 14 patients treated with thalidomide. The basic characteristics of the included studies are shown in Table 1. Among the included studies, one study had a low risk of bias (2), and three studies had a high risk of bias (20, 22, 31). The results of risk of bias assessments are shown in Supplementary Figures S1, S2. For the outcomes of pruritus relief, somnolence, and dizziness, the quality of the evidence was moderate. The quality of evidence assessed by GRADE is presented in Supplementary Table S1.

### 3.2 Pruritus relief of all drugs for up patients receiving hemodialysis

A total of 871 patients in 11 studies were included to assess different drugs for pruritus relief among patients receiving hemodialysis. The drugs involved cromolyn sodium, difelikefalin, gabapentin, hydroxyzine, melatonin, montelukast, nalbuphine, nalfurafine, nicotinamide, sertraline, and placebo. Of the drugs, hydroxyzine and gabapentin had a direct comparison, and all other drugs had a direct comparison with placebo. The thicker connecting lines and larger circles for gabapentin and placebo indicated that there was more literature and larger sample sizes for direct comparisons of these two drugs (Supplementary Figure S3).

**Figure 2:**
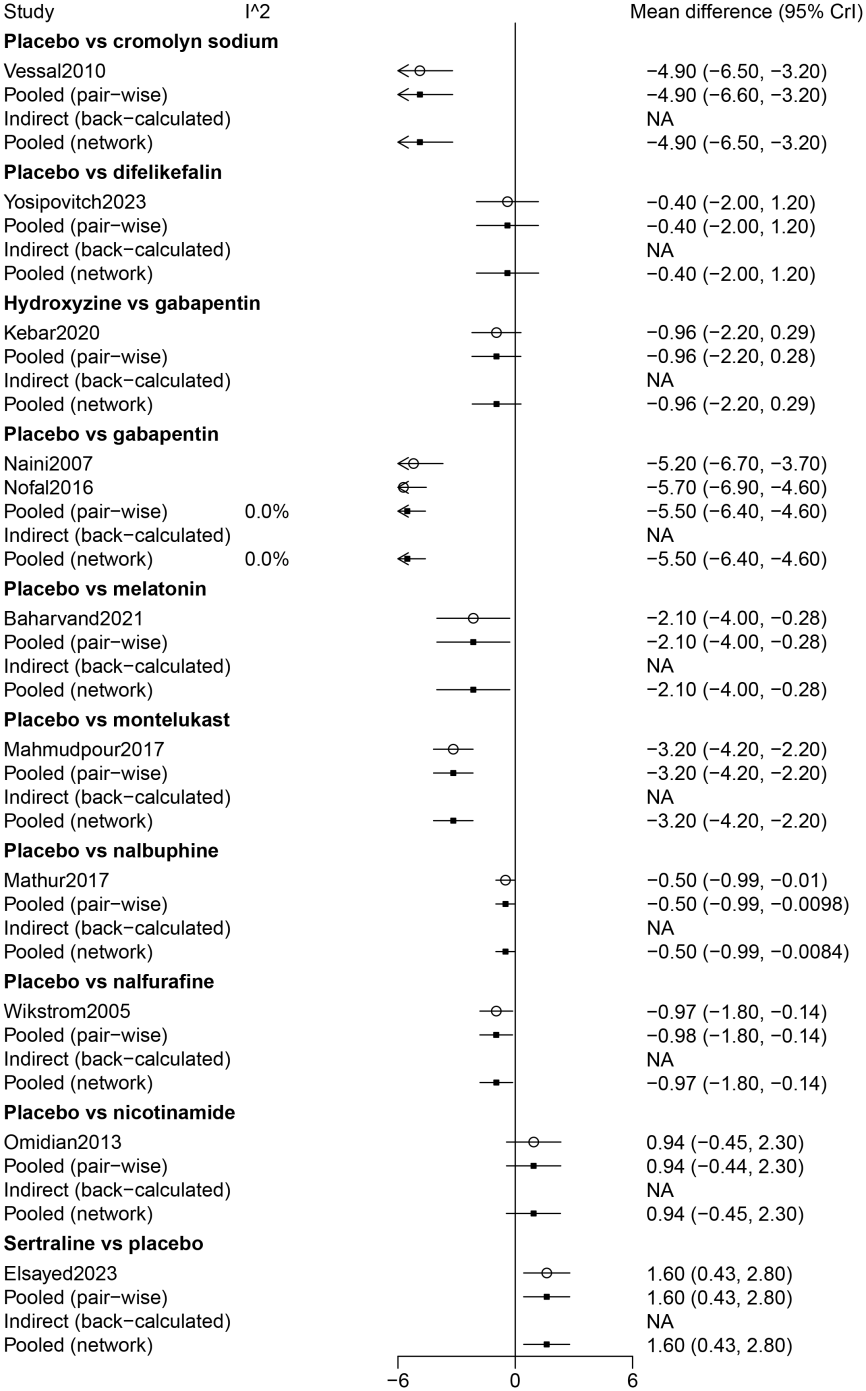
Forest plot of the comparison of all drugs in pruritus relief for UP patients receiving hemodialysis.

The findings of the direct comparison showed that compared with cromolyn sodium (WMD: −4.90, 95% CrI: −6.60, −3.20), gabapentin (WMD: −5.50, 95% CrI: −6.40, −4.60), melatonin (WMD: −2.1, 95% CrI: −4.0, −0.28), montelukast (WMD: −3.20, 95% CrI: −4.20, −2.20), nalbuphine (WMD: −0.50, 95% CrI: −0.99, −0.0098), nalfurafine (WMD: −0.98, 95% CrI: −1.80, −0.14), placebo was inferior in pruritus relief among patients receiving hemodialysis. In direct comparisons with placebo, sertraline was significantly more effective than placebo in pruritus relief among patients receiving hemodialysis (WMD: 1.60, 95% CrI: 0.43, 2.80). The findings of the direct comparison of drugs for pruritus relief are shown in Figure 2.

**Figure 3:**
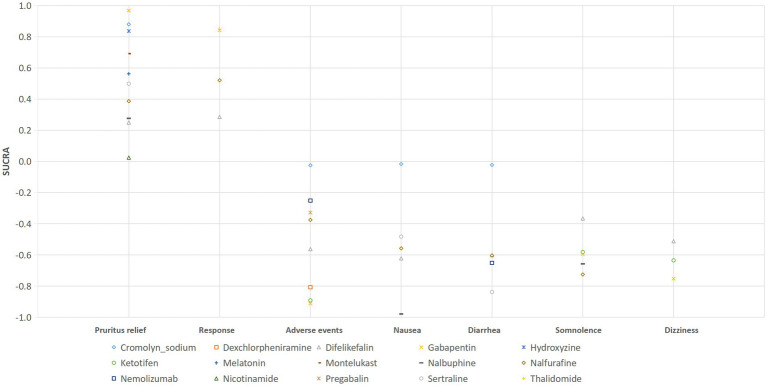
Ranking probabilities of the comparison of effects and safety of all drugs for UP patients receiving hemodialysis.

The results of the network meta-analysis showed that difelikefalin resulted in less pruritus relief than cromolyn sodium (WMD: −4.48, 95% CrI: −6.78, −2.17). Compared with difelikefalin, hydroxyzine was more effective in pruritus relief (WMD: 4.17, 95% CrI: 1.95, 6.39). By comparison with cromolyn sodium, melatonin had less pruritus relief (WMD: −2.73, 95% CrI: −5.24, −0.23). Melatonin was inferior to gabapentin in pruritus relief (WMD: −3.38, 95% CrI: −5.46, −1.29) (Table 2). According to the ranking table and the SUCRA, the most effective drug for pruritus relief was gabapentin, followed by cromolyn sodium, hydroxyzine, montelukast, melatonin, sertraline, nalfurafine, nalbuphine, difelikefalin, and nicotinamide (Table 3; Figure 3).

**Table 2:** League table of efficacy and safety of all drugs for UP patients receiving hemodialysis.

Pruritus relief											
	Cromolyn sodium	Difelikefalin	Gabapentin	Hydroxyzine	Melatonin	Montelukast	Nalbuphine	Nalfurafine	Nicotinamide	Placebo	Sertraline
Cromolyn sodium	Cromolyn sodium	−4.48 (−6.78, −2.17)	0.65 (−1.25, 2.56)	−0.31 (−2.58, 1.98)	−2.73 (−5.24, −0.23)	−1.70 (−3.66, 0.26)	−4.37 (−6.12, −2.63)	−3.91 (−5.78, −2.04)	−5.81 (−7.98, −3.64)	−4.87 (−6.54, −3.21)	−3.27 (−5.31, −1.22)
Difelikefalin	4.48 (2.17, 6.78)	Difelikefalin	5.13 (3.29, 6.96)	4.17 (1.95, 6.39)	1.75 (−0.70, 4.19)	2.78 (0.90, 4.65)	0.10 (−1.56, 1.76)	0.57 (−1.22, 2.36)	−1.33 (−3.42, 0.76)	−0.40 (−1.98, 1.18)	1.21 (−0.76, 3.18)
Gabapentin	−0.65 (−2.56, 1.25)	−5.13 (−6.96, −3.29)	Gabapentin	−0.96 (−2.21, 0.29)	−3.38 (−5.46, −1.29)	−2.35 (−3.72, −0.99)	−5.02 (−6.07, −3.98)	−4.55 (−5.80, −3.31)	−6.46 (−8.12, −4.80)	−5.53 (−6.44, −4.60)	−3.92 (−5.41, −2.42)
Hydroxyzine	0.31 (−1.98, 2.58)	−4.17 (−6.39, −1.95)	0.96 (−0.29, 2.21)	Hydroxyzine	−2.42 (−4.85, 0.00)	−1.40 (−3.26, 0.46)	−4.07 (−5.70, −2.44)	−3.59 (−5.37, −1.83)	−5.50 (−7.58, −3.42)	−4.57 (−6.13, −3.01)	−2.96 (−4.91, −1.00)
Melatonin	2.73 (0.23, 5.24)	−1.75 (−4.19, 0.7)	3.38 (1.29, 5.46)	2.42 (0, 4.85)	Melatonin	1.03 (−1.09, 3.14)	−1.65 (−3.58, 0.28)	−1.18 (−3.22, 0.87)	−3.08 (−5.4, −0.77)	−2.14 (−4.02, −0.28)	−0.54 (−2.74, 1.66)
Montelukast	1.7 (−0.26, 3.66)	−2.78 (−4.65, −0.9)	2.35 (0.99, 3.72)	1.4 (−0.46, 3.26)	−1.03 (−3.14, 1.09)	Montelukast	−2.67 (−3.79, −1.55)	−2.20 (−3.52, −0.89)	−4.11 (−5.82, −2.40)	−3.17 (−4.18, −2.16)	−1.56 (−3.11, −0.02)
Nalbuphine	4.37 (2.63, 6.12)	−0.1 (−1.76, 1.56)	5.02 (3.98, 6.07)	4.07 (2.44, 5.7)	1.65 (−0.28, 3.58)	2.67 (1.55, 3.79)	Nalbuphine	0.47 (−0.50, 1.44)	−1.44 (−2.89, 0.03)	−0.50 (−0.99, −0.01)	1.11 (−0.17, 2.38)
Nalfurafine	3.91 (2.04, 5.78)	−0.57 (−2.36, 1.22)	4.55 (3.31, 5.8)	3.59 (1.83, 5.37)	1.18 (−0.87, 3.22)	2.2 (0.89, 3.52)	−0.47 (−1.44, 0.5)	Nalfurafine	−1.90 (−3.51, −0.29)	−0.97 (−1.81, −0.14)	0.64 (−0.81, 2.08)
Nicotinamide	5.81 (3.64, 7.98)	1.33 (−0.76, 3.42)	6.46 (4.8, 8.12)	5.5 (3.42, 7.58)	3.08 (0.77, 5.4)	4.11 (2.4, 5.82)	1.44 (−0.03, 2.89)	1.9 (0.29, 3.51)	Nicotinamide	0.94 (−0.45, 2.31)	2.54 (0.73, 4.36)
Placebo	4.87 (3.21, 6.55)	0.39 (−1.18, 1.98)	5.53 (4.6, 6.45)	4.57 (3.01, 6.13)	2.15 (0.28, 4.02)	3.17 (2.16, 4.18)	0.5 (0.01, 0.99)	0.97 (0.13, 1.81)	−0.93 (−2.31, 0.44)	Placebo	1.61 (0.43, 2.79)
Sertraline	3.27 (1.22, 5.31)	−1.21 (−3.18, 0.76)	3.92 (2.42, 5.41)	2.96 (1, 4.91)	0.54 (−1.66, 2.74)	1.56 (0.02, 3.11)	−1.11 (−2.38, 0.17)	−0.64 (−2.08, 0.81)	−2.54 (−4.36, −0.73)	−1.61 (−2.79, −0.44)	Sertraline
Response											
	Difelikefalin	Gabapentin	Nalfurafine	Placebo	Thalidomide						
Difelikefalin	Difelikefalin	2.84 (1.48, 6.96)	1.35 (0.89, 2.11)	0.69 (0.59, 0.79)	3.91 (0.75, 104.82)						
Gabapentin	0.35 (0.14, 0.67)	Gabapentin	0.47 (0.18, 1.02)	0.24 (0.10, 0.45)	1.37 (0.21, 38.27)						
Nalfurafine	0.74 (0.47, 1.12)	2.11 (0.98, 5.5)	Nalfurafine	0.51 (0.33, 0.75)	2.90 (0.53, 78.62)						
Placebo	1.45 (1.26, 1.69)	4.13 (2.19, 10.05)	1.96 (1.33, 3.01)	Placebo	5.79 (1.10, 142.20)						
Thalidomide	0.26 (0.01, 1.33)	0.73 (0.03, 4.75)	0.34 (0.01, 1.9)	0.18 (0.01, 0.91)	Thalidomide						
Adverse events											
	Cromolyn sodium	Dexchlorpheniramine	Difelikefalin	Gabapentin	Ketotifen	Nalfurafine	Nemolizumab	Placebo	Pregabalin		
Cromolyn sodium	Cromolyn sodium	91.59 (5.01, 7378.64)	9.59 (1.59, 225.67)	127.55 (7.9, 9487.12)	130.05 (6.11, 10877.50)	8.42 (1.39, 199.64)	7.48 (1.2, 177.45)	8.39 (1.38, 215.60)	7.52 (0.11, 822.65)		
Dexchlorpheniramine	0.01 (0, 0.2)	Dexchlorpheniramine	0.12 (0.00, 0.88)	1.39 (0.65, 3.24)	1.4 (0.33, 5.92)	0.11 (0.00, 0.78)	0.10 (0.00, 0.71)	0.11 (0.00, 0.76)	0.09 (0.00, 0.59)		
Difelikefalin	0.1 (0, 0.63)	8.08 (1.13, 233.62)	Difelikefalin	11.14 (1.92, 303.80)	11.55 (1.29, 367.66)	0.88 (0.75, 1.04)	0.78 (0.58, 1.06)	0.87 (0.80, 0.93)	0.7 (0.02, 27.74)		
Gabapentin	0.01 (0, 0.13)	0.72 (0.31, 1.55)	0.09 (0, 0.52)	Gabapentin	1.00 (0.30, 3.32)	0.08 (0.00, 0.46)	0.07 (0.00, 0.42)	0.08 (0.003, 0.45)	0.06 (0.0023, 0.34)		
Ketotifen	0.01 (0, 0.16)	0.71 (0.17, 3)	0.09 (0, 0.78)	1 (0.3, 3.33)	Ketotifen	0.08 (0.00, 0.69)	0.07 (0.00, 0.62)	0.07 (0.00, 0.67)	0.06 (0.00, 0.51)		
Nalfurafine	0.12 (0.01, 0.72)	9.22 (1.28, 266.12)	1.14 (0.97, 1.33)	12.71 (2.17, 348.42)	13.16 (1.46, 419.34)	Nalfurafine	0.89 (0.67, 1.19)	0.99 (0.85, 1.12)	0.8 (0.02, 31.74)		
Nemolizumab	0.13 (0.01, 0.83)	10.41 (1.42, 302.97)	1.29 (0.94, 1.73)	14.34 (2.4, 392.61)	14.86 (1.61, 478.61)	1.13 (0.84, 1.49)	Nemolizumab	1.11 (0.82, 1.48)	0.9 (0.02, 36.17)		
Placebo	0.12 (0.01, 0.73)	9.34 (1.31, 269.37)	1.16 (1.07, 1.25)	12.88 (2.22, 350.99)	13.35 (1.49, 424.09)	1.01 (0.89, 1.18)	0.9 (0.68, 1.22)	Placebo	0.81 (0.02, 32.08)		
Pregabalin	0.13 (0, 8.81)	11.46 (1.69, 328.85)	1.43 (0.04, 55.81)	15.7 (2.96, 417.01)	16.33 (1.95, 501.53)	1.25 (0.03, 48.92)	1.11 (0.03, 43.67)	1.24 (0.03, 48.04)	Pregabalin		
Nausea											
	Cromolyn sodium	Difelikefalin	Nalbuphine	Nalfurafine	Placebo	Sertraline					
Cromolyn sodium	Cromolyn sodium	12.63 (1.77, 382.13)	154.91 (9.36, 13303.16)	14.56 (0.77, 1278.24)	7.40 (1.17, 202.73)	10.17 (0.99, 365.98)					
Difelikefalin	0.08 (0, 0.57)	Difelikefalin	10.49 (1.71, 291.12)	0.99 (0.13, 29.45)	0.61 (0.36, 1.02)	0.78 (0.20, 3.27)					
Nalbuphine	0.01 (0, 0.11)	0.1 (0, 0.58)	Nalbuphine	0.09 (0.00, 3.89)	0.06 (0.0022, 0.33)	0.07 (0.00, 0.67)					
alfurafine	0.07 (0, 1.29)	1.01 (0.03, 7.62)	10.76 (0.26, 456.84)	Nalfurafine	0.62 (0.02, 4.31)	0.76 (0.02, 8.48)					
Placebo	0.13 (0, 0.85)	1.63 (0.98, 2.79)	16.95 (3.07, 462.98)	1.62 (0.23, 45.77)	Placebo	1.27 (0.36, 4.92)					
Sertraline	0.1 (0, 1.01)	1.28 (0.31, 5.06)	13.87 (1.5, 439.04)	1.31 (0.12, 43.85)	0.79 (0.2, 2.81)	Sertraline					
Diarrhea											
	Cromolyn sodium	Difelikefalin	Nalfurafine	Nemolizumab	Placebo	Sertraline					
Cromolyn sodium	Cromolyn sodium	11.36 (1.44, 347.52)	13.66 (0.98, 608.32)	16.66 (0.89, 965.98)	5.91 (0.84, 170.4)	37.5 (1.77, 3244.93)					
Difelikefalin	0.09 (0, 0.69)	Difelikefalin	1.11 (0.23, 8.71)	1.33 (0.18, 17.05)	0.55 (0.34, 0.87)	2.86 (0.36, 75.91)					
Nalfurafine	0.07 (0, 1.02)	0.9 (0.11, 4.41)	Nalfurafine	1.15 (0.22, 9.67)	0.50 (0.06, 2.25)	2.6 (0.16, 90.58)					
Nemolizumab	0.06 (0, 1.12)	0.75 (0.06, 5.44)	0.87 (0.11, 4.65)	Nemolizumab	0.41 (0.03, 2.73)	2.19 (0.09, 91.05)					
Placebo	0.16 (0.01, 1.19)	1.81 (1.14, 2.93)	2 (0.44, 15.05)	2.4 (0.35, 29.83)	Placebo	5.00 (0.69, 154.78)					
Sertraline	0.03 (0, 0.56)	0.35 (0.01, 2.77)	0.38 (0.01, 6.42)	0.46 (0.01, 11.26)	0.19 (0.01, 1.45)	Sertraline					
Somnolence											
	Difelikefalin	Gabapentin	Ketotifen	Nalbuphine	Nalfurafine	Placebo					
Difelikefalin	Difelikefalin	2.18 (0.23, 64.30)	2.23 (0.16, 82.60)	2.65 (0.36, 76.81)	3.46 (0.49, 102.41)	0.56 (0.29, 1.01)					
Gabapentin	0.46 (0.02, 4.36)	Gabapentin	0.99 (0.25, 3.94)	1.24 (0.03, 58.98)	1.62 (0.04, 79.40)	0.25 (0.01, 2.17)					
Ketotifen	0.45 (0.01, 6.44)	1.01 (0.25, 4.02)	Ketotifen	1.25 (0.02, 74.11)	1.63 (0.03, 99.25)	0.25 (0.01, 3.31)					
Nalbuphine	0.38 (0.01, 2.74)	0.81 (0.02, 35.34)	0.8 (0.01, 45)	Nalbuphine	1.31 (0.03, 56.42)	0.21 (0.01, 1.36)					
Nalfurafine	0.29 (0.01, 2.03)	0.62 (0.01, 27.32)	0.61 (0.01, 34.17)	0.76 (0.02, 32.84)	Nalfurafine	0.16 (0.01, 0.99)					
Placebo	1.8 (0.99, 3.47)	3.92 (0.46, 112.13)	4.01 (0.3, 142.42)	4.74 (0.75, 132.89)	6.17 (1.01, 174.01)	Placebo					
Dizziness											
	Difelikefalin	Gabapentin	Ketotifen	Placebo							
Difelikefalin	Difelikefalin	2.01 (0.42, 15.95)	2.06 (0.04, 117.84)	0.57 (0.33, 0.93)							
Gabapentin	0.5 (0.06, 2.36)	Gabapentin	0.98 (0.03, 33.03)	0.28 (0.04, 1.22)							
Ketotifen	0.49 (0.01, 25.07)	1.02 (0.03, 38.23)	Ketotifen	0.27 (0.00, 13.77)							
Placebo	1.77 (1.08, 2.99)	3.53 (0.82, 26.95)	3.64 (0.07, 205.11)	Placebo							

UP, uremic pruritus.

**Table 3:** Ranking table of efficacy and safety of all drugs for UP patients receiving hemodialysis.

Pruritus relief											
	[,1]	[,2]	[,3]	[,4]	[,5]	[,6]	[,7]	[,8]	[,9]	[,10]	[,11]
Cromolyn sodium	0.247955	0.355995	0.347660	0.042190	0.005820	0.000375	0.000005	0.000000	0.000000	0.000000	0.000000
Difelikefalin	0.000000	0.000005	0.000040	0.001225	0.027660	0.086880	0.165140	0.197050	0.203250	0.226075	0.092675
Gabapentin	0.697445	0.285070	0.017425	0.000060	0.000000	0.000000	0.000000	0.000000	0.000000	0.000000	0.000000
Hydroxyzine	0.054075	0.347015	0.518905	0.069115	0.010380	0.000485	0.000025	0.000000	0.000000	0.000000	0.000000
Melatonin	0.000385	0.003940	0.024570	0.146830	0.480020	0.203875	0.077210	0.035250	0.017510	0.008480	0.001930
Montelukast	0.000140	0.007960	0.090845	0.723245	0.167495	0.010035	0.000255	0.000015	0.000000	0.000010	0.000000
Nalbuphine	0.000000	0.000000	0.000000	0.000000	0.000865	0.016350	0.137830	0.463955	0.352790	0.026385	0.001825
Nalfurafine	0.000000	0.000000	0.000005	0.000170	0.036985	0.194610	0.461035	0.222200	0.073875	0.009900	0.001220
Nicotinamide	0.000000	0.000000	0.000000	0.000005	0.000150	0.001065	0.004855	0.015175	0.035785	0.101480	0.841485
Placebo	0.000000	0.000000	0.000000	0.000000	0.000000	0.000000	0.000290	0.014280	0.300390	0.624530	0.060510
Sertraline	0.000000	0.000015	0.000550	0.017160	0.270625	0.486325	0.153355	0.052075	0.016400	0.003140	0.000355
Response											
	[,1]	[,2]	[,3]	[,4]	[,5]						
Difelikefalin	0.000000	0.005765	0.129955	0.864280	0.000000						
Gabapentin	0.386375	0.589920	0.023465	0.000240	0.000000						
Nalfurafine	0.006705	0.144265	0.772820	0.076090	0.000120						
Placebo	0.000000	0.000000	0.000000	0.017535	0.982465						
Thalidomide	0.606920	0.260050	0.073760	0.041855	0.017415						
Adverse events											
	[,1]	[,2]	[,3]	[,4]	[,5]	[,6]	[,7]	[,8]	[,9]		
Cromolyn sodium	0.00005	0.00006	0.00019	0.00315	0.00320	0.00208	0.00382	0.15443	0.83304		
Dexchlorpheniramine	0.12695	0.26249	0.58940	0.00825	0.00221	0.00334	0.00694	0.00041	0.00003		
Difelikefalin	0.00099	0.00342	0.02030	0.50222	0.42250	0.04575	0.00478	0.00006	0.00000		
Gabapentin	0.40014	0.49877	0.10008	0.00035	0.00042	0.00020	0.00006	0.00000	0.00000		
Ketotifen	0.47174	0.23374	0.27838	0.00574	0.00146	0.00257	0.00578	0.00059	0.00003		
Nalfurafine	0.00007	0.00051	0.00247	0.03755	0.26244	0.40466	0.24179	0.05003	0.00051		
Nemolizumab	0.00007	0.00031	0.00157	0.03123	0.08153	0.11487	0.40738	0.35689	0.00617		
Placebo	0.00000	0.00022	0.00108	0.00668	0.20039	0.41386	0.29967	0.07749	0.00063		
Pregabalin	0.00001	0.00051	0.00656	0.40486	0.02586	0.01269	0.02980	0.36011	0.15961		
Nausea											
	[,1]	[,2]	[,3]	[,4]	[,5]	[,6]					
Cromolyn sodium	0.00002	0.00162	0.00419	0.00979	0.04562	0.93878					
Difelikefalin	0.00133	0.32997	0.46362	0.18987	0.01491	0.00032					
Nalbuphine	0.90193	0.09384	0.00380	0.00044	0.00000	0.00000					
Nalfurafine	0.08964	0.35654	0.13946	0.11893	0.26053	0.03492					
Placebo	0.00000	0.00353	0.12722	0.44431	0.41855	0.00640					
Sertraline	0.00710	0.21451	0.26172	0.23668	0.26041	0.01959					
Diarrhea											
	[,1]	[,2]	[,3]	[,4]	[,5]	[,6]					
Cromolyn sodium	0.00113	0.00359	0.00722	0.014885	0.04144	0.931735					
Difelikefalin	0.053655	0.27088	0.316305	0.351355	0.00759	0.000215					
Nalfurafine	0.11715	0.279545	0.27013	0.1836	0.133955	0.01562					
Nemolizumab	0.22534	0.28657	0.19235	0.133965	0.139255	0.02252					
Placebo	0.000025	0.00479	0.086725	0.250325	0.633325	0.02481					
Sertraline	0.6027	0.154625	0.12727	0.06587	0.044435	0.0051					
Somnolence											
	[,1]	[,2]	[,3]	[,4]	[,5]	[,6]					
Difelikefalin	0.008215	0.063710	0.186955	0.254385	0.465275	0.021460					
Gabapentin	0.133570	0.270130	0.254170	0.175430	0.112720	0.053980					
Ketotifen	0.188395	0.241560	0.204980	0.143950	0.098360	0.122755					
Nalbuphine	0.292470	0.214900	0.167220	0.185950	0.091425	0.048035					
Nalfurafine	0.377350	0.209390	0.178010	0.154520	0.060300	0.020430					
Placebo	0.000000	0.000310	0.008665	0.085765	0.171920	0.733340					
Dizziness											
	[,1]	[,2]	[,3]	[,4]							
Difelikefalin	0.121910	0.301475	0.567755	0.008860							
Gabapentin	0.403925	0.473985	0.098495	0.023595							
Ketotifen	0.473860	0.195450	0.092945	0.237745							
Placebo	0.000305	0.029090	0.240805	0.729800							

UP, uremic pruritus.

### 3.3 Drug response of all drugs for up patients receiving hemodialysis

A total of seven studies, involving 1,611 patients, were included to assess the drug response of UP treatment for patients receiving hemodialysis. All drugs were compared directly with placebo. The thicker lines and larger circles between difelikefalin and placebo indicated a larger literature and sample size for direct comparisons between the two (Supplementary Figure S4).

**Figure 4:**
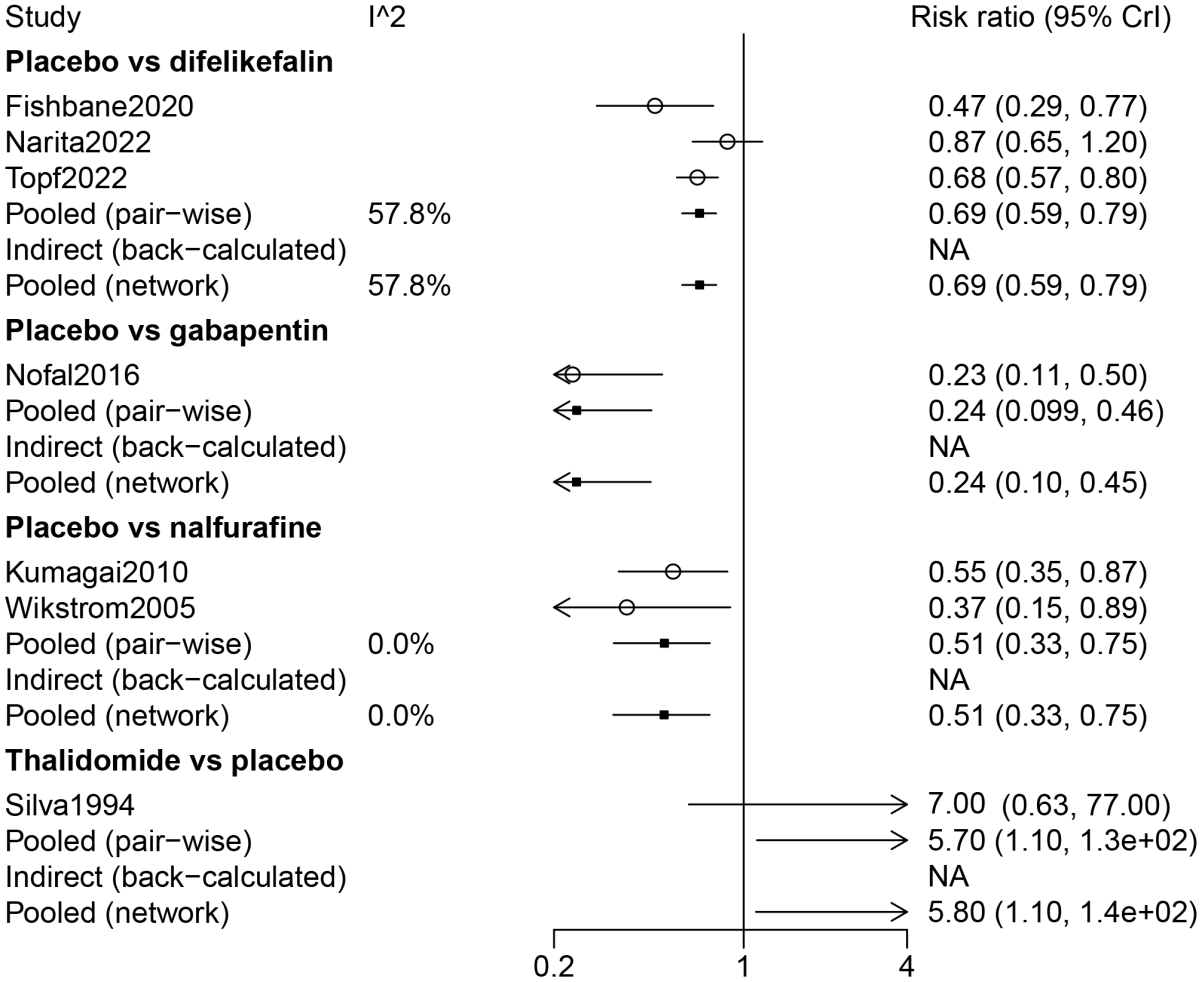
Forest plot of the comparison of all drugs in drug response for UP patients receiving hemodialysis.

By direct comparison with difelikefalin (RR: 0.69, 95% CrI: 0.59, 0.79), gabapentin (RR: 0.24, 95% CrI: 0.099, 0.46), and nalfurafine (RR: 0.51, 95% CrI: 0.33, 0.75), placebo was inferior in response to UP treatment for patients receiving hemodialysis. Thalidomide was more effective on drug response than placebo (RR: 5.70, 95% CrI: 1.10, 130) (Figure 4).

The results of the network meta-analysis indicated that compared with difelikefalin, gabapentin was superior in the drug response of UP treatment (RR: 2.84, 95% CrI: 1.48, 6.96); compared with difelikefalin, placebo was less responsive (RR: 0.69, 95% CrI: 0.59, 0.79) (Table 2). By comparison with gabapentin and nalfurafine, placebo was inferior in drug response (Table 2). According to the ranking table and the SUCRA, the order of drug response was thalidomide, gabapentin, nalfurafine, and difelikefalin (Table 3; Figure 3).

### 3.4 Risk of adverse events of all drugs for up patients receiving hemodialysis

The risk of adverse events was investigated in 12 studies, including 2,150 patients. Placebo was directly comparable to cromolyn sodium, gabapentin, nalfurafine, nemolizumab, and difelikefalin. Gabapentin was in direct comparison with ketotifen, dexchlorpheniramine, and pregabalin. Direct comparisons were found for nalfurafine with nemolizumab. The thicker connecting lines and larger circles for placebo and nalfurafine indicate a larger literature and larger sample size for a direct comparison of these two (Supplementary Figure S5).

**Figure 5:**
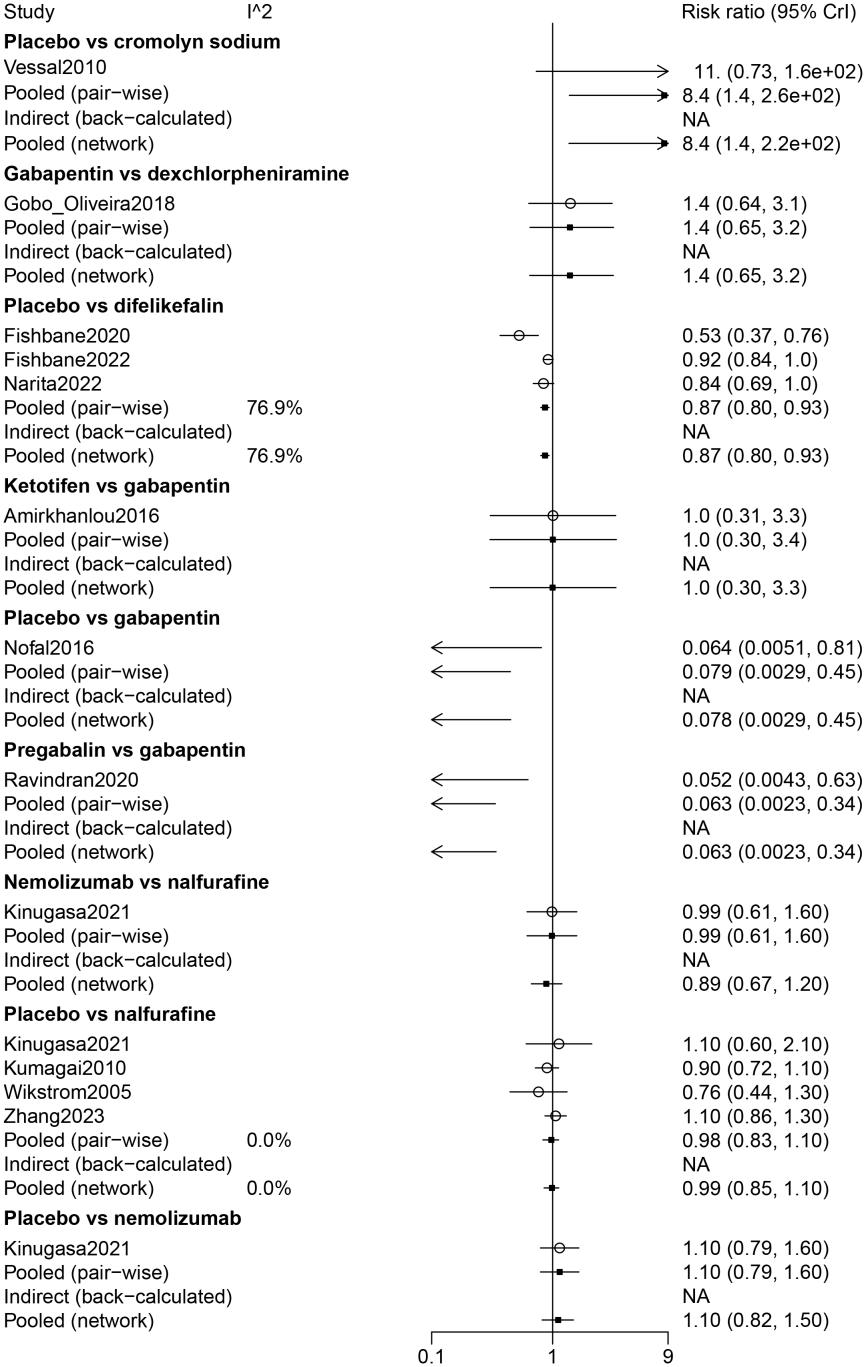
Forest plot of the comparison of all drugs in adverse events for UP patients receiving hemodialysis.

The results of direct comparisons showed that placebo had more adverse effects than cromolyn sodium (RR: 8.40, 95% CrI: 1.40, 260). Nevertheless, compared with difelikefalin (RR: 0.87, 95% CrI: 0.80, 0.93) and gabapentin (RR: 0.079, 95% CrI: 0.0029, 0.45), the placebo had less adverse effects. Pregabalin had less adverse effects than gabapentin (RR: 0.063, 95% CrI: 0.0023, 0.34) (Figure 5).

The results of the network meta-analysis suggested that dexchlorpheniramine (RR: 91.59, 95% CrI: 5.01, 7378.64) and difelikefalin (RR: 9.59, 95% CrI: 1.59, 225.67) had more adverse effects than cromolyn sodium. Less adverse effects were found in difelikefalin by comparison with dexchlorpheniramine (RR: 0.12, 95% CrI: 0.00, 0.88) (Table 2). Adverse events in descending order were gabapentin, ketotifen, dexchlorpheniramine, difelikefalin, nalfurafine, pregabalin, nemolizumab, and cromolyn sodium (Table 3; Figure 3).

### 3.5 Nausea of all drugs for up patients receiving hemodialysis

A total of seven studies examined nausea of UP treatment for patients receiving hemodialysis. Cromolyn sodium, difelikefalin, nalbuphine, nalfurafine, and sertraline were all directly compared with placebo. The thicker connecting lines and larger circles for difelikefalin and placebo demonstrated that more literature and larger sample sizes were available for direct comparisons of these two (Supplementary Figure S6).

**Figure 6:**
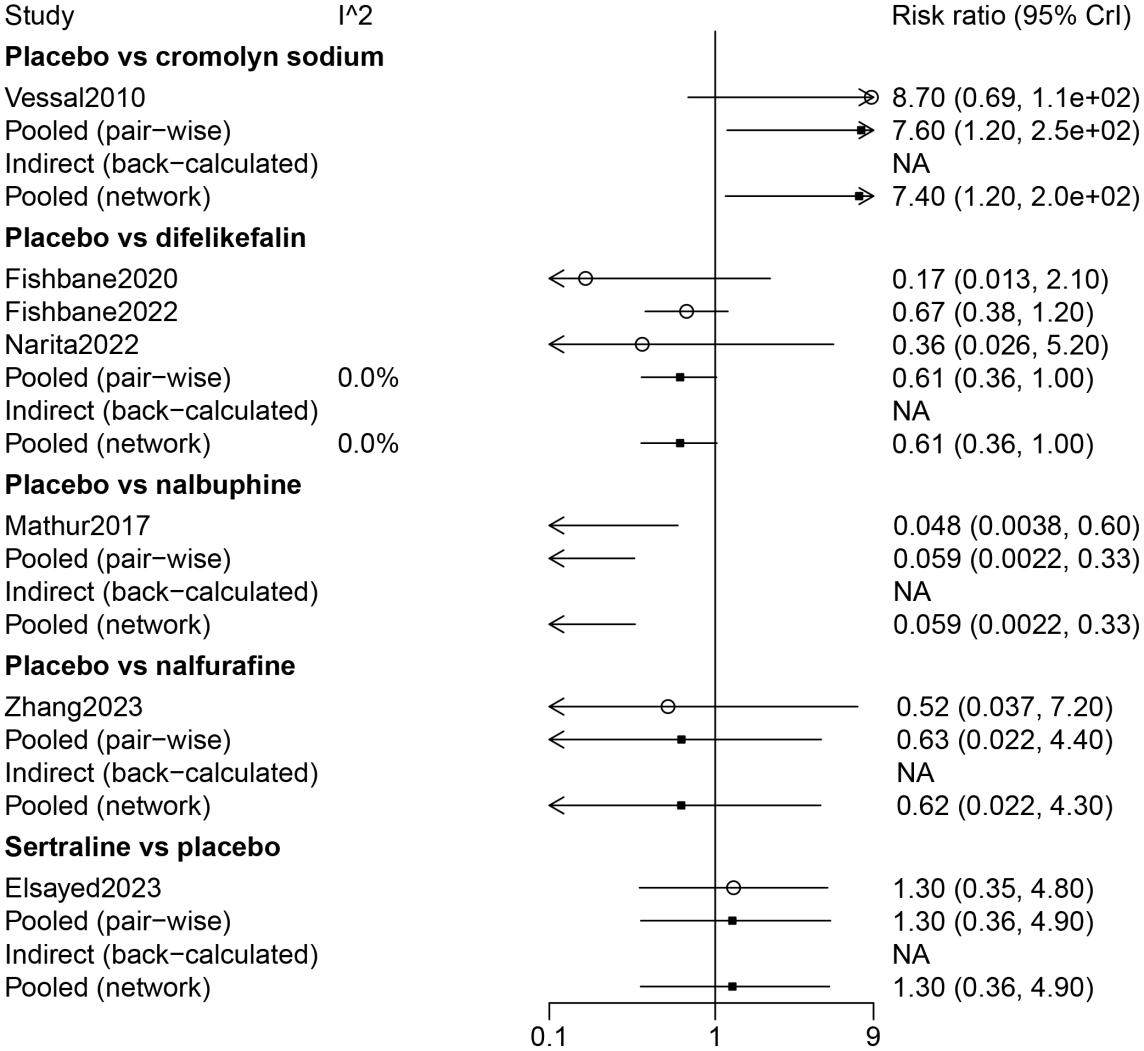
Forest plot of the comparison of all drugs in nausea for UP patients receiving hemodialysis.

The results of direct comparisons showed that patients with a placebo had more nausea than patients with cromolyn sodium (RR: 7.60, 95% CrI: 1.20, 250.0). However, less nausea was observed in patients with a placebo compared with patients with nalbuphine (RR: 0.059, 95% CrI: 0.0022, 0.33) (Figure 6).

The results of the network meta-analysis indicated that by comparison with patients who received cromolyn sodium, patients who received difelikefalin, nalbuphine, and placebo had more nausea. Patients treated with nalbuphine had more nausea than difelikefalin (RR: 10.49, 95% CrI: 1.71, 291.12). By comparison with nalbuphine, placebo (RR: 0.06, 95% CrI: 0.002, 0.33) and sertraline (RR: 0.07, 95% CrI: 0.00, 0.67) had less nausea. Nausea occurred in the order of nalbuphine, difelikefalin, nalfurafine, sertraline, and cromolyn sodium (Table 3; Figure 3).

### 3.6 Diarrhea of all drugs for up patients receiving hemodialysis

Diarrhea of UP treatment for patients receiving hemodialysis was assessed in six studies. Cromolyn sodium, difelikefalin, nalfurafine, nemolizumab, and sertraline were directly compared with placebo. There were direct comparisons between nalfurafine and nemolizumab. The thicker lines and larger circles between nalfurafine and placebo indicated a larger literature and sample size for direct comparisons between the two (Supplementary Figure S7).

**Figure 7:**
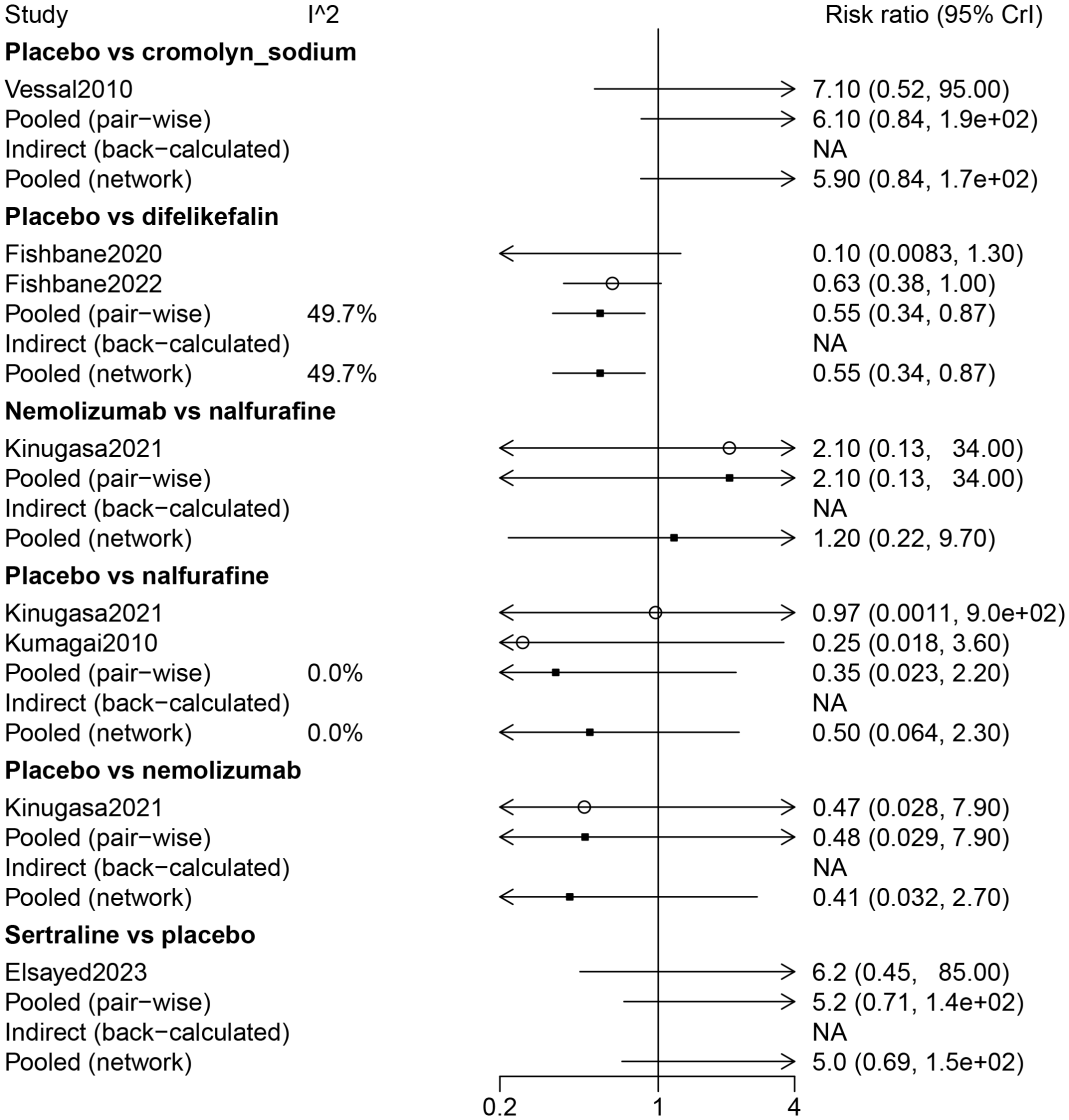
Forest plot of the comparison of all drugs in diarrhea for UP patients receiving hemodialysis.

The results of the direct comparison showed that the placebo had less diarrhea compared with difelikefalin (RR: 0.55, 95% CrI: 0.34, 0.87) (Figure 7). The results of the network meta-analysis suggested that patients receiving difelikefalin (RR: 11.36, 95% CrI: 1.44, 347.52) and sertraline (RR: 37.5, 95% CrI: 1.77, 3244.93) had more diarrhea compared with patients receiving cromolyn sodium. Patients who received a placebo had less diarrhea than patients who received difelikefalin (RR: 0.55, 95% CrI: 0.34, 0.87). The order of risk of diarrhea was sertraline > nemolizumab > nalfurafine > difelikefalin > cromolyn sodium (Table 3; Figure 3).

### 3.7 Somnolence of all drugs for up patients receiving hemodialysis

A total of 2,083 patients from seven studies were included to assess the somnolence of UP treatment for patients receiving hemodialysis. Ketotifen was directly compared with gabapentin. Nalfurafine, nalbuphine, gabapentin, and difelikefalin were directly compared with placebo (Supplementary Figure S8).

**Figure 8:**
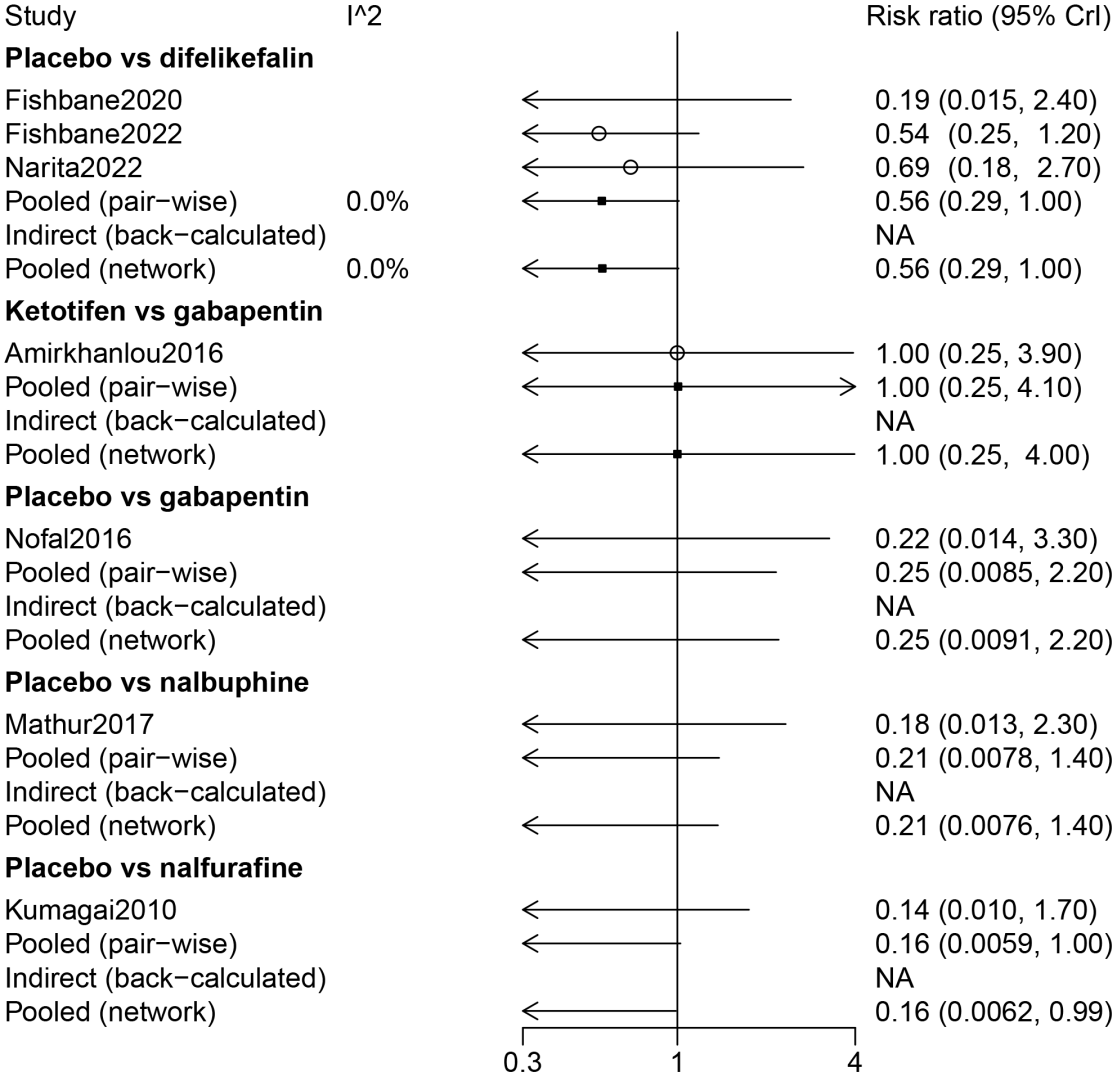
Forest plot of the comparison of all drugs in somnolence for UP patients receiving hemodialysis.

Results of the forest plot showed that there was no statistical difference among the drugs in somnolence (Figure 8). The results of the network meta-analysis suggested that patients who received a placebo had less risk of somnolence than patients who received nalfurafine (RR: 0.16, 95% CrI: 0.01, 0.99) (Table 2). According to the ranking table and the SUCRA, the drugs with high to low risk of somnolence were ranked as nalfurafine, nalbuphine, gabapentin, ketotifen, and difelikefalin (Table 3; Figure 3).

### 3.8 Dizziness of all drugs for up patients receiving hemodialysis

Dizziness of UP treatment was assessed in six studies. Gabapentin was directly compared with ketotifen and placebo. There was a direct comparison between difelikefalin and placebo (Supplementary Figure S9).

**Figure 9:**
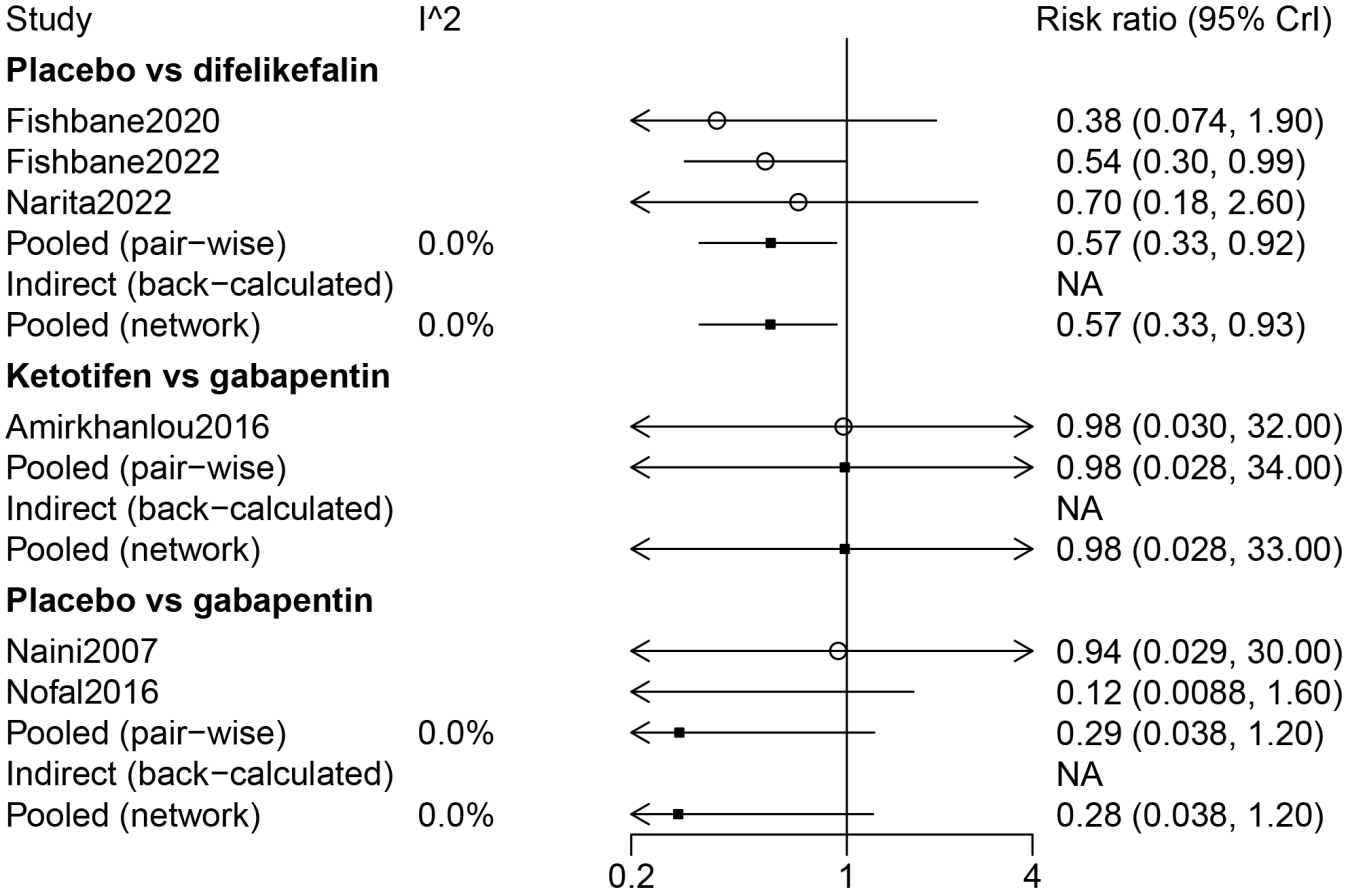
Forest plot of the comparison of all drugs in dizziness for UP patients receiving hemodialysis.

The results of direct comparison and the results of the network meta-analysis showed that a lower risk of dizziness was observed in patients with placebo compared with patients with difelikefalin (RR: 0.57, 95% CrI: 0.33, 0.92) (Figure 9). According to the ranking table and the SUCRA, the risk of dizziness in drugs was ranked as gabapentin > ketotifen > difelikefalin (Table 3; Figure 3).

## 4 Discussion

Based on the RCTs, the present network meta-analysis analyzed and ranked the efficacy and safety of different drugs in the treatment of UP in hemodialysis patients. Our primary findings suggested that gabapentin, followed by cromolyn sodium, was superior to pruritus relief for treating UP among patients receiving hemodialysis. Thalidomide and gabapentin were more likelihood to have a higher drug response for treating UP among patients receiving hemodialysis. A higher risk of adverse events and dizziness was more likely to be observed in patients who were treated with gabapentin. Lower rankings of adverse events, nausea, and diarrhea were found in patients who received cromolyn sodium.

Gabapentin is an anticonvulsant drug that was initially developed and approved as an adjunctive therapy for the treatment of partial seizures (36) and may control pruritus of neuropathic origin (37). In the present meta-analysis, gabapentin was effective for pruritus relief and had a better response to the UP. In the RCT, Gobo-Oliveira et al. reported that gabapentin alleviated pruritus symptoms (11). In an RCT and review of literature, gabapentin is a promising and well-tolerated treatment option for patients with UP (29). In the double-blind clinical trial conducted in patients older than 18 years who had undergone hemodialysis, the author found that gabapentin is an effective agent in treating UP (38). The clinical benefit observed with the use of gabapentin for UP can be explained by the fact that gabapentin impedes transmitting nociceptive sensations to the brain, thus also suppressing pruritus (39). A study focusing on the recent advances in the treatment of UP observed that gabapentin appears to be the most evidence-based, widely available UP treatment, as long as care is taken with dosing and monitoring of side effects (40). Dizziness is the most common side effect of gabapentin found in this meta-analysis. Amirkhanlou et al. reported that 19.2% of patients receiving gabapentin suffered from drowsiness and dizziness, but no serious side effects were observed (19). The present meta-analysis indicated gabapentin is a promising drug for treating UP, and an advanced understanding of the pathological itching components and the mechanism of action of gabapentin may aid in the selection of therapeutic agents and adjustment of dosage according to the period of itch presented by the patient, offering satisfactory treatment.

The present network revealed that cromolyn sodium also had better pruritus relief, as well as the lowest risks of nausea, diarrhea, and adverse events. In a previous study, the author reported that cromolyn sodium might offer an alternative therapy for patients with refractory UP (41). In a double-blind placebo-controlled study, cromolyn sodium can significantly reduce the severity of pruritus in hemodialysis patients (34). In a study assessing the effects of different pharmacological treatments for preventing or treating pruritus in adult palliative care patients, cromolyn sodium relieved UP participants from pruritus by 2.94 points on the VAS (42). We also observed that thalidomide had the highest drug response. The study by Sharma et al. proposed that thalidomide can be an alternative or combination antipruritic treatment for chronic refractory pruritus patients who do not obtain enough relief from conservative therapy (43). Given the limited number of studies, large, rigorous, multiarm RCTs to assess the efficacy and safety of cromolyn sodium and thalidomide for UP patients receiving hemodialysis are urgently needed.

Our study holds significant clinical importance. First, this network meta-analysis design allows for the comparison of multiple systemic drug treatments within the same analysis, providing a comprehensive overview of the available treatment options. This is particularly valuable in UP, where there is no consensus on the most effective treatment, and options may vary widely in terms of efficacy, safety, and mechanism of action. Second, by systematically reviewing and analyzing data from RCTs, the study provides evidence-based insights into the relative efficacy and safety of different systemic drugs. This information can guide healthcare providers in selecting the most appropriate treatment for their patients, potentially leading to more effective management of UP. Third, the inclusion of safety data in the analysis is crucial, as hemodialysis patients often have multiple comorbidities and may be more susceptible to adverse drug reactions. Understanding the safety profile of each treatment option enables clinicians to make more informed choices, balancing efficacy with the risk of side effects. Fourth, by synthesizing existing research, the study may also reveal areas where the evidence is lacking or inconsistent, highlighting the need for further research. This can stimulate additional studies focused on under-researched drugs or lead to the development of new therapeutic options for UP. Fifth, the clinical significance of this study lies in its potential to improve patient outcomes. By providing a clearer understanding of the most effective and safe treatments for uremic pruritus, the study can help alleviate this debilitating symptom, improving the overall wellbeing and quality of life of hemodialysis patients.

The first advantage of this network meta-analysis is that the studies included were all RCTs. Second, we used Bayesian approaches, which can facilitate the integration of ancillary information regarding variables under study through prior probability distribution. However, this network meta-analysis also had limitations. First, most trials compared systemic drugs with placebos, and there were fewer trials that directly compared different drugs. Although network meta-analyses can statistically infer comparisons between treatments that have not been directly compared, the strength and reliability of these inferences are contingent on the network’s density and connectivity. Fewer direct comparisons can lead to wider confidence intervals and less certainty in the indirect comparisons made, potentially affecting the robustness of the meta-analysis findings. Second, due to the limitations of the included studies, the sample size of some drugs was relatively small. Small sample sizes in clinical trials can significantly limit the statistical power of an analysis. This limitation makes it more challenging to detect the true differences or effects of the drugs being studied. Third, a few patients in the included studies were still routinely taking anti-pruritus medication at the time of the intervention. The concurrent use of anti-pruritus medications during the intervention period introduces a confounding variable that can obscure the true effect of the systemic drugs being studied. This makes it difficult to attribute any observed changes in pruritus severity solely to the intervention drug, as the effect could be influenced or moderated by the other medications. Fourth, these included studies only involved papers published in the English language; although publication bias was analyzed, the lack of papers published in another language except English limited more in-depth analyses than were reported here.

## 5 Conclusion

Our results suggest considering gabapentin when facing a patient suffering from UP among hemodialysis patients. However, further studies are required to examine and rank the efficacy and safety of drug treatment for UP in hemodialysis patients.

## Data availability statement

The raw data supporting the conclusions of this article will be made available by the authors, without undue reservation.

## Author contributions

XZ: Writing – review & editing, Writing – original draft, Project administration, Methodology, Funding acquisition, Conceptualization. HS: Writing – review & editing, Software, Investigation, Formal analysis, Data curation. WL: Writing – review & editing, Writing – original draft, Project administration, Data curation, Conceptualization.
