# Supplementary material for: Efficacy and safety of different systemic drugs in the treatment of uremic pruritus among hemodialysis patients: a network meta-analysis based on randomized clinical trials
Source: Front Med (Lausanne). 2024 Apr 5;11:1334944. doi: 10.3389/fmed.2024.1334944 (PMC11026555; doi:10.3389/fmed.2024.1334944)
Supplement: Supplementary file 1 [file Table_1.DOCX]

**Supplementary Table 1 The quality of evidence assessed by GRADE**

| **Certainty assessment** | | | | | | | **Certainty** | **Importance** |
| --- | --- | --- | --- | --- | --- | --- | --- | --- |
| **N of studies** | **Study design** | **Risk of bias** | **Inconsistency** | **Indirectness** | **Imprecision** | **Other considerations** |  |  |
| **Pruritus relief** | | | | | | |  |  |
| 11 | Randomised trials | Serious^a^ | Not serious | Not serious | Not serious | None | ⨁⨁⨁◯ Moderate | Critical |
| **Response** | | | | | | |  |  |
| 7 | Randomised trials | Serious^a^ | Serious^b^ | Not serious | Not serious | None | ⨁⨁◯◯ Low | Critical |
| **Adverse events** | | | | | | |  |  |
| 12 | Randomised trials | Serious^a^ | Serious^b^ | Not serious | Serious^c^ | None | ⨁◯◯◯ Very low | Important |
| **Nausea** | | | | | | |  |  |
| 7 | Randomised trials | Serious^a^ | Not serious | Not serious | Serious^c^ | None | ⨁⨁◯◯ Low | Important |
| **Diarrhea** | | | | | | |  |  |
| 6 | Randomised trials | Serious^a^ | Not serious | Not serious | Serious^c^ | None | ⨁⨁◯◯ Low | Important |
| **Somnolence** | | | | | | |  |  |
| 7 | Randomised trials | Serious^a^ | Not serious | Not serious | Not serious | None | ⨁⨁⨁◯ Moderate | Important |
| **Dizziness** | | | | | | |  |  |
| 6 | Randomized trials | Serious^a^ | Not serious | Not serious | Not serious | None | ⨁⨁⨁◯ Moderate | Important |

Notes: GRADE: Grading of Recommendations Assessment, Development and Evaluation; CI: confidence interval; a. random method and blind method are not clear; b. large heterogeneity; c. some controls had larger CIs.
