# Supplementary figures and images for: Efficacy and safety of different systemic drugs in the treatment of uremic pruritus among hemodialysis patients: a network meta-analysis based on randomized clinical trials
Source: Front Med (Lausanne). 2024 Apr 5;11:1334944. doi: 10.3389/fmed.2024.1334944 (PMC11026555; doi:10.3389/fmed.2024.1334944)

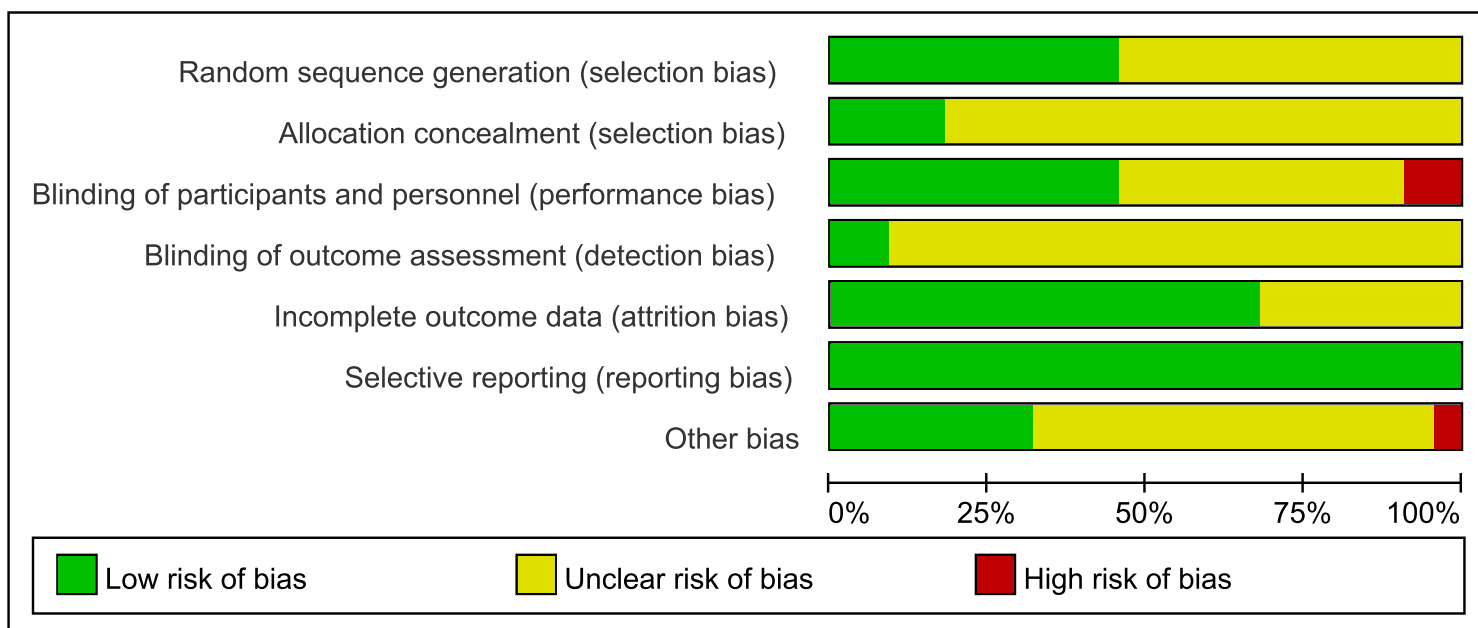

Supplement: Supplementary file 2 [file Image_1.pdf]

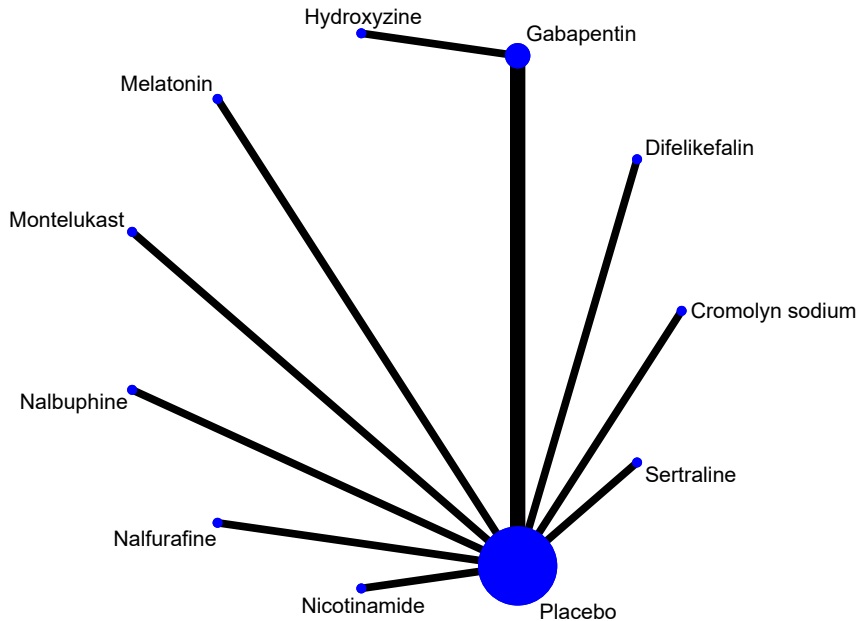

Supplement: Supplementary file 4 [file Image_3.pdf]

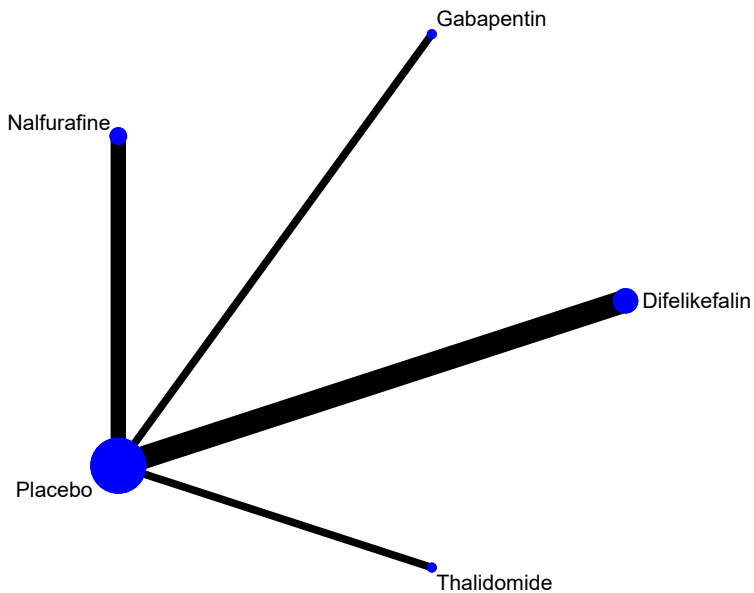

Supplement: Supplementary file 5 [file Image_4.pdf]

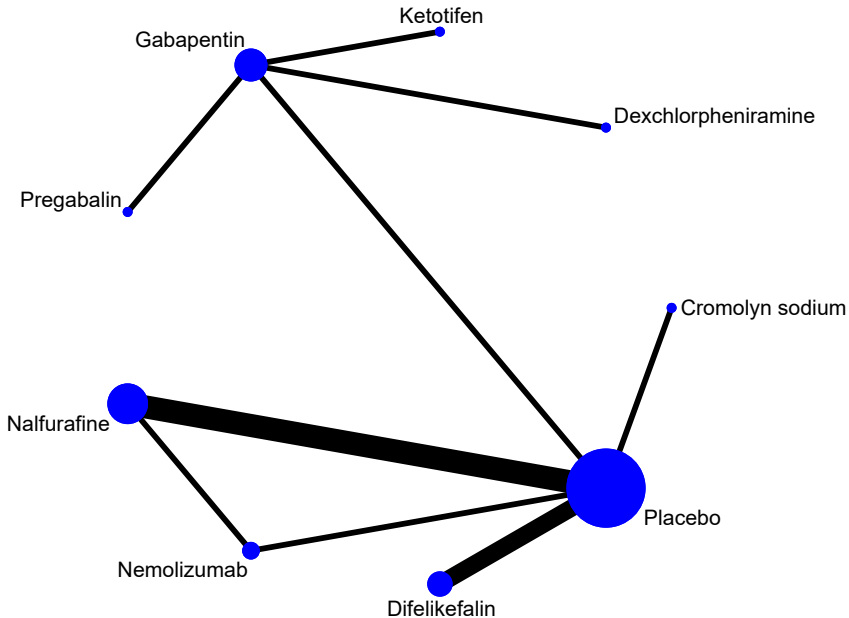

Supplement: Supplementary file 6 [file Image_5.pdf]

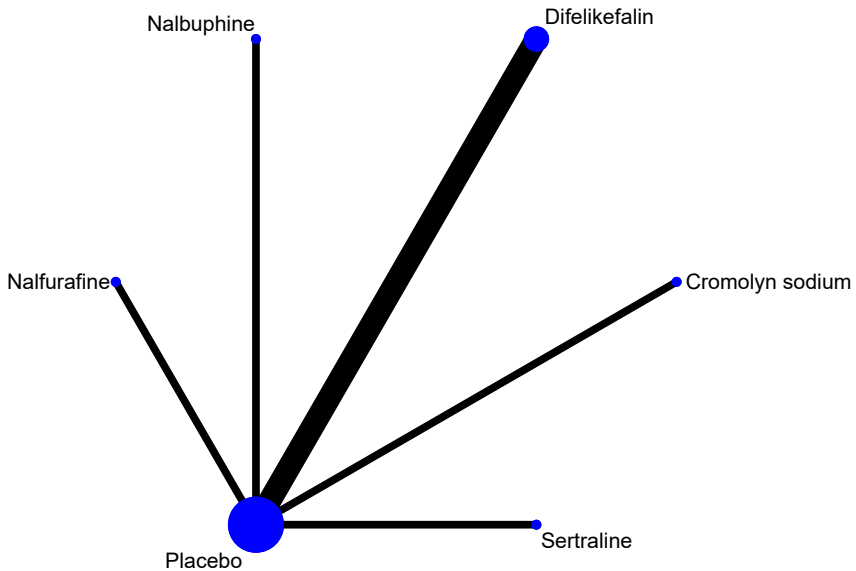

Supplement: Supplementary file 7 [file Image_6.pdf]

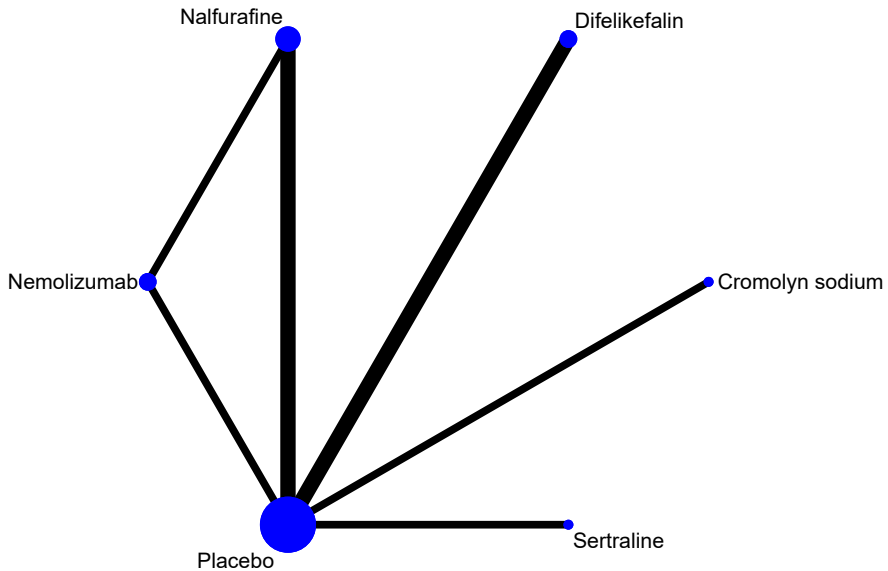

Supplement: Supplementary file 8 [file Image_7.pdf]

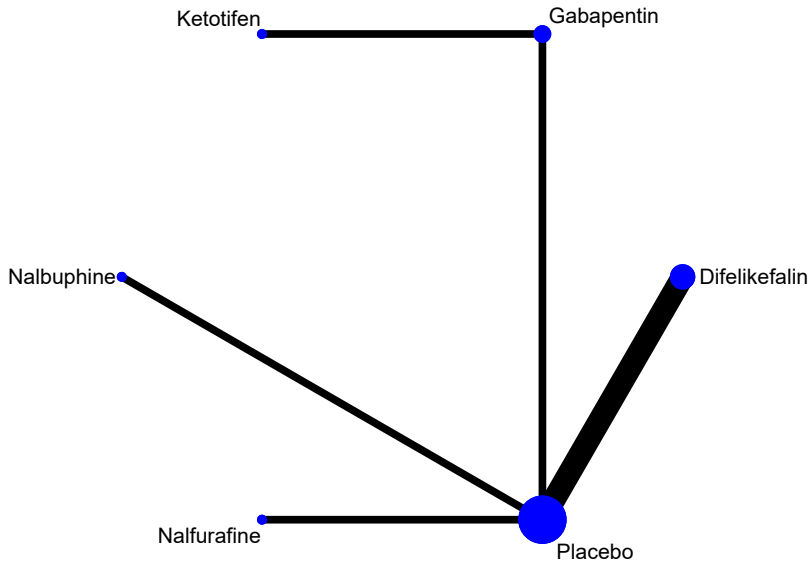

Supplement: Supplementary file 9 [file Image_8.pdf]

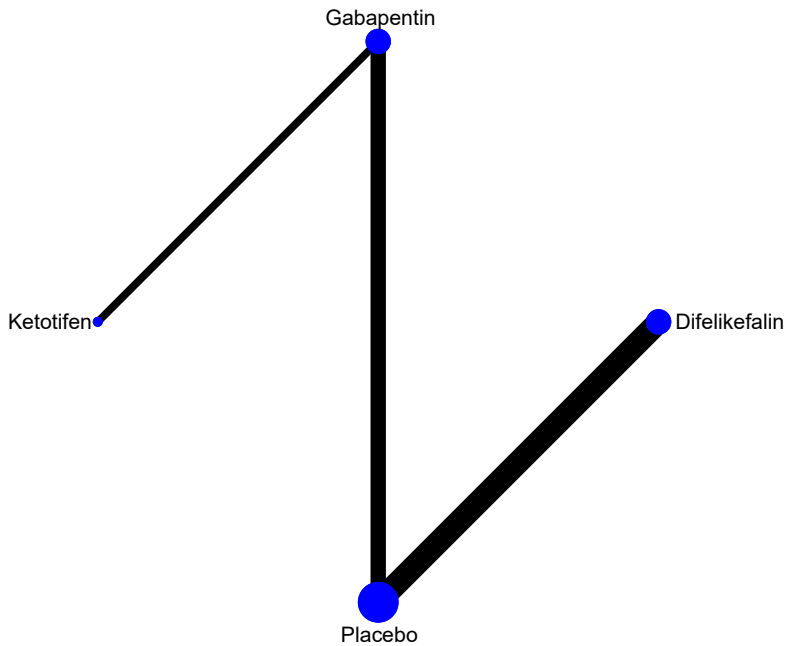

Supplement: Supplementary file 10 [file Image_9.pdf]
