# Supplementary material for: Efficacy and safety of different systemic drugs in the treatment of uremic pruritus among hemodialysis patients: a network meta-analysis based on randomized clinical trials
Source: Front Med (Lausanne). 2024 Apr 5;11:1334944. doi: 10.3389/fmed.2024.1334944 (PMC11026555; doi:10.3389/fmed.2024.1334944)
Supplement: Supplementary file 3 [file Image_2.pdf]

	Random sequence generation (selection bias)	Allocation concealment (selection bias)	Blinding of participants and personnel (performance bias)	Blinding of outcome assessment (detection bias)	Incomplete outcome data (attrition bias)	Selective reporting (reporting bias)	Other bias
Amirkhanlou2016	?	?	?	?	?	+	?
Baharvand2021	+	?	+	?	?	+	−
Elsayed2023	+	+	+	+	+	+	+
Fishbane2020	+	?	+	?	+	+	?
Fishbane2022	?	?	?	?	+	+	?
Gobo Oliveira2020	+	+	+	+	+	+	?
Kebar2020	?	?	?	?	+	+	+
Kinugasa2021	?	?	−	?	+	+	?
Kumagai2010	?	?	?	?	+	+	?
Mahmudpour2017	?	?	+	?	+	+	+
Mathur2017	+	+	+	?	?	+	?
Naini2007	?	?	+	?	?	+	+
Narita2022	?	?	?	?	+	+	?
Nofal2016	+	?	?	?	+	+	+
Omidian2013	+	?	?	?	+	+	+
Ravindran2020	?	?	−	?	?	+	?
Silva1994	?	?	?	?	?	+	?
Topf2022	?	?	?	?	+	+	?
Vessal2010	+	?	+	?	?	+	+
Wikstrom2005	?	?	?	?	+	+	?
Yosipovitch2023	+	?	+	?	+	+	?
Zhang2023	+	+	+	?	+	+	?
